# Associations between body size, nutrition and socioeconomic position in early life and the epigenome: A systematic review

**DOI:** 10.1371/journal.pone.0201672

**Published:** 2018-08-10

**Authors:** Jane Maddock, Wahyu Wulaningsih, Juan Castillo Fernandez, George B. Ploubidis, Alissa Goodman, Jordana Bell, Diana Kuh, Rebecca Hardy

**Affiliations:** 1 MRC Unit for Lifelong Health and Ageing, Institute of Cardiovascular Science, University College London, London, United Kingdom; 2 Department of Twin Research and Genetic Epidemiology, King's College London, London, United Kingdom; 3 Centre for Longitudinal Studies, UCL Institute of Education, University College London, London, United Kingdom; Erasmus MC, NETHERLANDS

## Abstract

**Background:**

Body size, nutrition and socioeconomic position (SEP) in early life have been associated with a wide range of long-term health effects. Epigenetics is one possible mechanism through which these early life exposures can impact later life health. We conducted a systematic review examining the observational evidence for the impact of body size, nutrition and SEP in early life on the epigenome in humans.

**Methods:**

This systematic review is registered with the PROSPERO database (registration number: CRD42016050193). Three datasets were simultaneously searched using Ovid and the resulting studies were evaluated by at least two independent reviewers. Studies measuring epigenetic markers either at the same time as, or after, the early life exposure and have a measure of body size, nutrition or SEP in early life (up to 12 years), written in English and from a community-dwelling participants were included.

**Results:**

We identified 90 eligible studies. Seventeen of these papers examined more than one early life exposure of interest. Fifty six papers examined body size, 37 nutrition and 17 SEP. All of the included papers examined DNA methylation (DNAm) as the epigenetic marker. Overall there was no strong evidence for a consistent association between these early life variables in DNAm which may be due to the heterogeneous study designs, data collection methods and statistical analyses.

**Conclusions:**

Despite these inconclusive results, the hypothesis that the early life environment can impact DNAm, potentially persisting into adult life, was supported by some studies and warrants further investigation. We provide recommendations for future studies.

## Introduction

Substantial evidence from the field of life course epidemiology has supported a relationship between physical and social exposures across the entire life course and later life health [1]. Rapid growth and development that occurs in early life marks a sensitive period during which external factors can influence an individual’s later life health [2–4] Evidence has accumulated for the importance of nutrition and growth in utero and early postnatal life on a wide range of health and ageing outcomes such as cardiometabolic and bone health [5]. Childhood socioeconomic position (SEP) has also been found to be associated with a wide range of later life health outcomes [6, 7].

Exposures in early life must impact the organism in order for their effects to manifest after a long latency period. The biological, behavioural and psychosocial mechanisms linking these earlier life exposures with later life health are complex [1, 3]. Epigenetics is one possible mechanism [3, 8–10]. Epigenetics refers to processes that regulate gene expression but do not change the underlying DNA sequence. These tissue and cell-specific processes include DNA methylation (DNAm), histone modification, other changes to chromatin structure, and post-transcriptional control [3]. Genetic variation, stochastic events as well as the environment have been shown to influence the epigenome [11]. Since these epigenetic processes can persist during mitosis, it is feasible that early life exposures influencing the epigenome may have a phenotypic manifestation in later life [9].

A number of early life exposures have been investigated in relation to epigenetics. Animal studies have made a convincing case for the role of nutrition during fetal and early neonatal growth on epigenetics [12, 13]. DNA or histone methylation in offspring in these studies has been shown to be particularly susceptible to maternal dietary intake of folate, vitamin B6 (pyridoxine), vitamin B12 (cobalamin), vitamin B2 (riboflavin), choline and methionine. These nutrients are involved in one-carbon metabolism, influencing the amount of available S-adenosylmethionine and co-enzymes which are required for methylation [14]. In human studies, participants who were affected by the Dutch Famine provide evidence for the lasting impact of severe caloric restriction during particular periods of gestation [12, 15]. The role of nutrition on epigenetics beyond this fetal and early neonatal period is less studied [12]. Growth and body size in early life are related to nutrition, and indeed there is also evidence for predominantly cross-sectional associations between birth weight, childhood and adolescence BMI/obesity, body composition and DNA methylation from human studies [15]. The small number of human studies also suggest a role for early life SEP on DNA methylation [15].

Since this is a relatively new and rapidly developing area of research, most evidence examining the epigenetic effect of these key early life factors have come from animal and exploratory studies incorporating a variety of early life exposures and applying different analytical methods. In 2015 Demetriou *et al*. conducted a non-systematic review of the evidence for early-life nutrition, SEP and overweight/obesity on DNA methylation [15]. In 2017, Hartwig *et al*. systematically reviewed the literature of the effects of breastfeeding on DNA methylation [16]. To the best of our knowledge, there has been no comprehensive systematic review of the potential effects of the key early life exposures of nutrition, body size and SEP on epigenetic processes. Therefore, the aim of this study was to systematically review the literature on the association between 1) body size and growth in early life 2) nutrition during pregnancy and early life 3) markers of SEP in early life on epigenetic processes in human studies. This will provide information on the potential for epigenetics to mediate the association between these early life exposures and later life health.

## Methods

This systematic review is registered with the PROSPERO database (registration number: CRD42016050193) and the protocol has been published in a peer-review journal [17].

### Eligibility criteria

We included studies that tested the association between any measure of (i) body size or growth in early life, (ii) nutrition during pregnancy or early life, or (iii) SEP in early life on epigenetics in human samples. We defined early life as 12 years and under to capture exposures during the pre-adolescent period including prenatal, infancy, early and middle childhood. We considered any indicator of DNAm or histone modification measured in any tissue as an outcome. Early life factors could be prospectively measured or recorded, or retrospectively recalled at later data collections. Eligible measures of body size were weight, height, BMI, and head circumference at birth or any stage in early life or change in any of these measurements. Nutrition included any measure of maternal nutrition, supplement use and/or diet during pregnancy, breastfeeding/formula, weaning practices and nutrition/diet of the child in early life measured using dietary questionnaires and/or objectively by nutritional biomarkers. Eligible measures of SEP included any recognised indicator of SEP within society, including occupation, education, income, occupational or social class, poverty, and household overcrowding, as defined by Krieger *et al*. [18].

Reviews, clinical trials, animal studies, studies assessing the effect of adulthood exposures on epigenetic markers and those assessing the epigenetic marker before the early life measure were excluded. Studies in samples with a specific clinical condition were excluded. Studies were only included if they were published in the English language in peer-reviewed journals.

### Search strategy

We performed a systematic review of the literature in March 2017. Using OvidSP as the database interface, a joint electronic search on MEDLINE and Embase was conducted. We searched BIOSIS database using ISI Web of Science. The search used free-text search terms (S1 Table) with truncations to allow for different spellings, proximity operators (‘adj’ in OvidSP, ‘NEAR’ in ISI Web of Science) and joined using Boolean logic (“AND”, “OR”). The reference lists’ of relevant reviews, all included papers and their ISI citation index (via Web of Science) was searched for studies meeting inclusion criteria. Given the extensive number of studies identified using these databases; we did not search grey literature. Eligible studies identified were combined with the electronic search results.

### Study selection and data extraction

All abstracts were screened independently for eligibility by two researchers (from JM, WW and RH). The full text of all potentially eligible papers was also double screened by JM, RH, WW and JCF and reasons for their exclusion were documented. Disagreements about the paper’s eligibility were resolved through discussion and if necessary, a third reviewer.

The following information was extracted from selected papers: citation details, study details (including type, country/region and sample size), participant details (including age and sex), and exposure and outcome details (including details on methods used). A free-text box for recording main findings was used because of the expected heterogeneous methods that will have been used.

The following aspects of the paper which may relate to the quality of each study were extracted: study type, methods used to measure epigenetics, statistical analysis (including adjustment of relevant confounders), recall bias such as prospective or retrospective measures of early life factors, and generalisability [19].

Due to the diversity in eligible studies in terms of methods used, a meta-analysis was not conducted [19]. Therefore, a narrative synthesis was undertaken [20].

## Results

**Fig 1:**
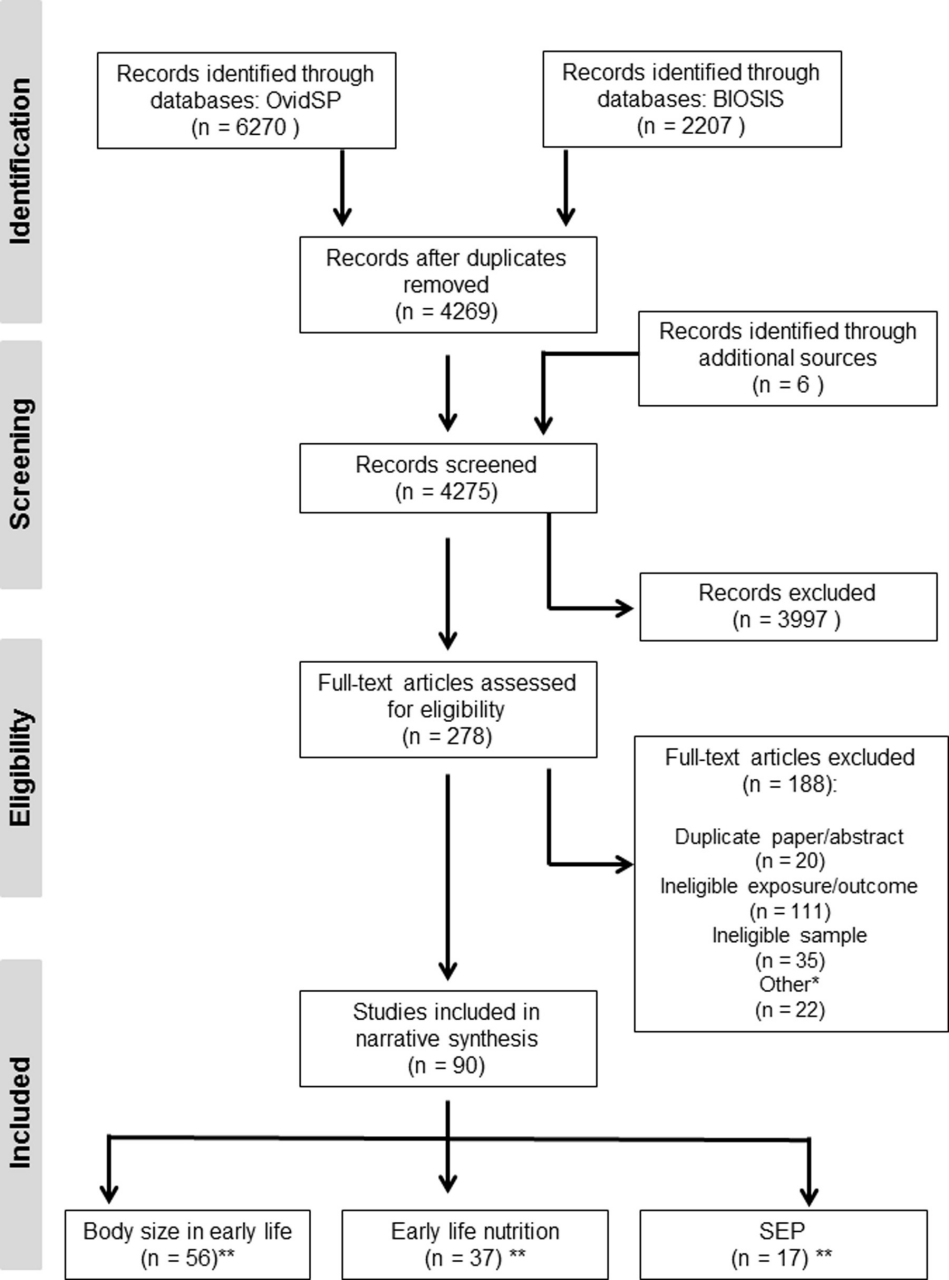
PRISMA Flow diagram of study selection. *Other includes: reviews, not peer reviewed, publication not found, randomised control trials, animal studies **N’s including overlapping studies (*n* = 17).

Overall we identified 90 eligible papers (Fig 1 and Tables 1–3). Seventeen of these papers examined more than one early life exposure of interest. All of the included papers examined DNAm as the epigenetic marker with none examining histone modifications. Results of each of these papers will be outlined below according to the main exposure of interest.

**Table 1:** Body size in early life and epigenetics. *(Organised by study design, *exposure*, *DNA methylation (epigenome wide*, *global methylation*, *imprinted genes*, *other genes)*.

First author (year), country	Cohort, N (% female)	Early life variable(mean age ± SD (age range)	DNA methylation	Tissue	Mean age at epigenetic measure±SD (age range)	Main result	Confounders
**CROSS-SECTIONAL**							
**Body size at birth**							
Engel (2014), Norway [21]	MoBa, 1,046 (46)	Birth weight,(Birth, GAD 96% 37-≥42w)	Infinium Human Methylation450 BeadChip	Cord blood	Birth (GAD 96% 37- ≥42w)	Adjusted mean difference (SE) in birthweight(g) per logit increase in methylation fraction of CpGs at Bonferroni significance *p*<0.05 (5x10^−2^): cg25953130(gene: *ARID5B*): −371.3 (58.1), *p* = 8.11×10^−5^ cg08005122(gene: NA):−493.4 (83.7), *p* = 1.82×10^−3^ cg20076442(gene: NA):−302.8 (51.6), *p* = 2.17×10^−3^ cg02863179(gene: *ARID5B*):−341.0 (59.3), *p* = 4.33×10^−3^ cg25124943(gene: NA):−480.2 (89.5), *p* = 3.96×10^−2^ cg00049440(gene: *KLF9*):−314.3 (58.7), *p* = 4.14×10^−2^ cg02194129(gene: X*RCC3*):726.6 (96.3), *p* = 2.24×10^−8^ cg17836177(gene: *PEBP4*):558.4 (93.1), *p* = 9.61×10^−4^ cg12798040(gene: X*RCC3*):326.3 (54.3), *p* = 1.21×10^−3^ cg00605777(gene:*SEMA4C*):728.3 (122.9), *p* = 1.50×10^−3^ cg14172849(gene: *XRCC3*):660.4 (112.6), *p* = 2.17×10^−3^ cg23127323(gene: *SPON2*):323.1 (56.7), *p* = 5.97×10^−3^ cg25162533(gene: NA):382.7 (68.3), p = 1.01×10^−2^ cg23369670(gene: *XRCC3*):557.3 (99.7), p = 1.12×10^−2^ cg17714703(gene: *UHRF1*):297.0 (54.1), p = 1.97×10^−2^ cg08420923(gene: *ANKRD11*):493.2 (91.5), *p* = 3.45×10^−2^ cg23237276(gene: NA):523.2 (97.7), *p* = 4.12×10^−2^ cg05993265(gene: *MFSD10)*:442.0 (82.6), *p* = 4.23×10^−2^ cg24693803(gene: NA):538.7 (101.1), *p* = 4.84×10^−2^	Child sex, maternal plasma cotinine, parity, maternal age, dietary folate (not including supplements), asthma, GAD, GAD^2^, pre-eclampsia, season of birth, leucocyte cell-type composition
Haworth (2014), UK [22]	Discovery cohort: 12 (NR)Investigation cohort: 110 (53.2)	BWP,(Birth, median GAD 39.4 (IQR:38.7–40.3w))	Discovery cohort Infinium Human Methylation450 BeadChip Investigation cohort*PM20D1*, *MIR886*, *SDHAP3*, *FGFR2* using pyrosequencing	Cord blood	Birth (GAD median: 39.4 (IQR:38.7–40.3w))	314 candidate genes with CpG sites associated with BWP. 27 of these genes had ≥2 CpGs associated with BWP. Authors focused on genes with a difference in methylation β-value ≥0.2 between low and high BWP groups. Four loci with ≥4 CpG sites were identified; *MIR886*, *PM20D1*, *SDHAP3*, *FGFR2*. *SDHAP3* was not validated using pyrosequencing and therefore not brought forward. Discovery cohort: Pearson’s R^2^ for BWP and methylation β-value: *PM20D1* (cg07167872): 0.48 (ranging 0.38–0.55 for other CpGs) *MIR886* (cg06536614): 0.62 *FGFR2* (cg18566515): 0.71 Investigation cohort: No association between *PM20D1* or *MIR886* with BWP Median methylation % of *FGFR2* across BWP groups *p* = 0.005, (*p* = 0.013 after correction for multiple testing): low (<15^th^): 25.7 medium (>40^th^—<60th): 24.0 and high (>85^th^): 36.5 *FGFR2* higher among high BWP (>85^th^) vs all others: cg18566515: p = 0.004 cg18566515 + 17 bp: *p* = 0.027 cg18566515 + 31 bp: *p* = 0.008 Proportion of cases with *FGFR2* methylation levels below 20%: High BWP group:7.1% vs. all other BMP groups: 33.7% This suggests that low methylation in *FGFR2* is associated with reduced risk of high birth weight	
Turan (2012), US [23]	70 (44.2)	Birth weight(Birth)	GoldenGate methylation array (1536 CpGs in 700 genes that were selected for their functions in cell growth, proliferation or embryonic development) & Infinium HumanMethylation450 BeadChip	Cord blood	Birth	Correlation (R^2^) between methylation of mechanism-based candidate genes and birth weight in GoldenGate CpGs (*n* = 22) & Infinium HumanMethylation450 BeadChip CpGs (*n* = 48) ***Gene***: *GoldenGate CpGs(R*^*2*^*)*; *Infinium CpGs*(*R*^*2*^*)*: ***IGF1*:** cg17084217(0.004), cg25163611(1.0x10^-4^); cg01305421(0.005) ***IGF1R*:** cg19714640(**0.097**), cg20742855(0.005); cg22375192(0.011), cg02166532(0.006) ***IGF2*:** cg10649864(0.007), cg17626526(0.040), cg17084217(0.011); cg02807948(0.049), cg13756879(4.0x10^-04^), cg20339650 (0.014), cg22956483(3.0x10^-04^), cg01305421(0.032) ***IGF2BP1*:** NA; cg06638433(0.005), cg13877465(0.019) ***IGF2BP2*:** NA; cg18234011(0.005), cg24450631(0.005) ***IGF2BP3*:** cg00508334(3.5x10^-05^), cg21413760(0.062); cg02860543(0.049), cg19042950(1.2x10^-05^) ***IGF2R*:** cg07148501(0.009), cg12721534(0.014); cg00230368(0.007), cg14556618(8.4x10^-05^) ***IGFBP1*:** cg20666158(0.015), cg23864854(0.048); cg05660795(0.033), cg27447599(0.021) ***IGFBP2*:** cg07828219(0.032), cg17207942(0.035); cg25854162(0.004), cg26187237(6.7x10^-05^) ***IGFBP3*:** cg12826145(0.023), cg14625938(0.001); cg04796162(0.014), cg06713098(0.027), cg08831744(0.001), cg15898840(0.026), cg22083798(0.029) ***GFBP4*:** cg03940014(0.054), cg22392383(0.018); cg00512374(0.008) ***IGFBP5*:** cg20419545(0.066), cg24617085(0.067); cg19008649(0.021), cg22467567(0.001) ***IGFBP6*:** cg00122038(0.009), cg22732012(0.072); cg01773854(0.051), cg08629913(0.024) ***IGFBP7*:** cg00431950(0.023), cg16546204(0.026); cg00884221(0.002), cg03876618 (3.3x10^-05^) ***INS*:** cg13349859(0.001), cg14426263(0.008); cg00613255(0.001), cg03366382(1.0E-04), cg13993218(0.003), cg25336198(0.005) ***INSR*:** cg05427477(0.002), cg19110381(0.072); NA ***PHLDA2*:** cg03637064(0.019), cg18242686(0.024),; cg04720330(4.0x10^-05^), cg11961618(0.039), cg14415214(0.001), cg21259253(4.0x10^-04^), cg26799802(3.0x10^-04^), cg00702231(0.019), cg07077459(**0.055**) ***PLAGL1*:** cg10923987(0.002), cg12757684(0.067); cg08263357(3.8x10^-06^), cg12757684(0.001), cg14161241(0.002), cg17895149(0.001), cg22378065(0.017), cg25350411(0.002), cg00613255(0.007), cg03366382(0.010) ***IGF2/H19*:** cg25871270(0.001), cg19731870(0.002); NA Methylation levels at these genes explained 26% (GoldenGate) or 46% (Illumina 450k) of birth weight trait variance. GoldenGate machine learning identified six genes(*APOE*, *MSX1*, *GRB10*, *PGRMC1*, *RGS14*, *SHMT2*) whose methylation level accounted for 78% of the variance in birth weight Infinium HumanMethylation450 BeadChip machine learning identified seven genes *(ATP6AP1*, *PRSS21*, *RCOR1*, *ANGPT4*, *CDK2*, *EVPL*, *NAT8L*) whose methylation level accounted for 70% of the variance in birth weight The combined model, using methylation levels at all 13 candidate genes identified in both experiments, explains 84% of the variance in birth weight in the sample of 48 individuals	GA
Adkins (2012), US [24]	CANDLE, 201(45.3)	Birth weight,(Birth, GAD 39.1±1 (36-41w))	Infinium Human Methylation27 BeadChip	Cord blood	Birth (GAD 39.1±1w)	No genome-wide significance for change in birth weight per increase in % methylation was reached for any CpG sites at Bonferroni correction *p* = 1.9x10^-6^ Top 10 methylated gene sites: *GALK1*: *p* = 0.0002 *GPR40*: *p* = 0.0002 *AQP12A*: *p* = 0.0002 *HSD3B2*: *p* = 0.0003 *TTLL2*: *p* = 0.0003 *BZRAP1*: *p* = 0.0004 *SNX6*: *p* = 0.0004 *TACR2*: *p* = 0.0007 *CUL7*: *p* = 0.0008 *HOXB2*: *p* = 0.0008	Newborn sex, maternal BMI, race, GA
Fryer (2011), UK [25]	12(92)	Birth weight,(Birth)	Infinium Human Methylation27 BeadChip	Cord blood	Birth	Two clusters were identified following unsupervised hierarchical clustering to identify underlying β-value-derived methylation across the samples. BWP was higher (*p* = 0.019) in cluster B. 304 CpGs associated with BWP (*p*<0.05, full results NR)	
Lee (2012), US [26]	THREE, 141 (47)	Birth weight, (Birth, 87% GAD ≥37w)	Genome-wide DMRs identified using microarray technique, CHARM 2.0	Cord blood	Birth	Average residual DNA methylation across top three DMRs associated with birth weight (kg) β (95%CI): *NFIX*: 2.86 (0.87,4.84) *RAPGE*:-2.89 (-4.43,-1.34) *MSRB*:-3.97 (-5.71,-2.22)	Surrogate variables estimated via SVA
Herbstman (2013), US [27],	CCCEH, 279 (53.4)	Birth weight, Birth length Ponderal Index, Head Circumference,(Birth)	Global methylation using Methylamp Global DNA Methylation Quantification Kit	Cord blood	Birth	Change in birth outcomes per increase in log-transformed DNA methylation (95% CI) *Birth weight (g)*: -28.45 (-72.06,15.16) *Birth length (cm)*: 0.05 (-0.25,0.35) *Ponderal Index (g/m*^*3*^*)*: -0.27 (-0.69,0.14) *Head Circumference (cm*): -0.13 (-0.26,0.006)	GA, plate, maternal height, pre-pregnancy BMI, maternal age at delivery, ethnicity, sex, public assistance, total polycyclic aromatic hydrocarbons and tobacco smoke, delivery mode (for head circumference)
Nomura (2014), US [28]	50 (42)	Birth weight, Head circumference, Hody length,(Birth)	Global methylation using LUMA	Cord blood	Birth	Association between global methylation (%) and birth outcomes in multivariate general linear model, β(SE), *p* *Birth weight*: 0.62(8.75), *p* = 0.94 *Head circumference*: 0.02 (0.05), *p* = 0.76 *Body length*: -0.02 (0.09), *p* = 0.85	Newborn sex, mother's education, welfare status, material status, ethnicity, GA
Haggarty (2013), UK [29]	1,073 (NR)	Birth weight, Crown heel length,(Birth, GAD 39±2w)	LINE-1 (4 CpGs), *IGF2* (4 CpGs), *PEG3* (7 CpGs), *SNRPN* (4 CpGs) using pyrosequencing	Cord blood	Birth (GAD 39±2w)	Change in early life variable per % increase methylation (average across CpGs). Coefficient (95% CI): *LINE-1* Birth weight(g): -13.55 (-27.18,0.09), *p* = 0.05 Crown heel length (cm): -0.03 (-0.09, 0.03), *p* = 0.33 *PEG3* Birth weight(g): 3.03 (-7.56,13.63), *p* = 0.58 Crown heel length (cm): 0.01 (-0.03,0.06), *p* = 0.51 *SNRPN* Birth weight(g): 1.90 (-5.24,9.05), *p* = 0.60 Crown heel length (cm): -0.005 (-0.03,0.03), *p* = 0.75 *IGF2* Birth weight(g): -6.08 (-12.22,0.06), *p* = 0.05 Crown heel length (cm): -0.02 (-0.04,0.01), *p* = 0.22	Newborn sex, GA
Michels (20122), US [30]	The Epigenetic Birth Cohort, 319 (48)	Birth weight, (Birth, 88% GAD ≥37w)	LINE-1 using pyrosequencing	Cord blood	Birth (88% GAD ≥37w)	Difference in % methylation (95% CI) *Birth weight (g)*: <2500: *n* = 29: -0.82(-1.42, -0.23), *p* = 0.007 2500–3999: *n* = 277 (ref) 4000+: *n* = 62–0.43 (-0.84, -0.03) *p* = 0.04	Maternal age at delivery, maternal ethnicity, maternal smoking prior to or during pregnancy, newborn sex, preterm birth
Nafee (2009), UK** [31]	24(NR)	Birth weight (birth)	LINE-1 using pyrosequencing	Cord blood	Birth	LINE-1 methylation associated with BWP *p* = 0.014, adjusted R^2^ = 0.211	
Burris (2013), Mexico [32]	219 (47.2)	Birth weight (Birth, GAD 39.1±1.1w)	*ICR1* (4 CpGs), *ICR2* (4 CpGs), *H19* (2 CpGs), LINE-1 (4 CpGs), *Alu* (3 CpGs), *NR3C1* (5 CpGs), *GCR* (1 CpG) using pyrosequencing	Cord blood	Birth (GAD39.1±1.1w)	Mean birth weight (g) difference (95% CI) with 1 SD increase in DNA methylation (average of each CpG site within each locus): *IGF2* regulatory complex *ICR1*: 3(-50,56) *ICR2*: -20(-72,32) *H19* promotor: 4(-49,57) Glucocorticoid receptor *GCR*:-1(-53,52) *NR3C1*:-39(-94,15) Repetitive elements *LINE-1*:16(-38,70) *Alu*:-8(-61,44)	GA, maternal age, second trimester maternal weight, parity, education, infant sex
Bouwland-Both (2013), The Netherlands [33]	Generation R, SGA: 69 (43) AGA (control): 471 (43)	Birth weight, (GA 40.3w)	*IGF2* DMR, *H19* promotor, *MTHFR* using mass-spectrometry based method	Cord blood	Birth (GA 40.3w)	Adjusted difference (95% CI) in % methylation for SGA vs. control. *IGF2* DMR: -1.07 (-1.93, -0.21), *p* = 0.015 *H19*: -0.27 (-0.94, 0.39), *p* = 0.42 *MTHFR* was found to be hypomethylated with limited variability between SGA and controls and no further analysis was conducted: median % methylation (90% range): 2.4 (1.5–3.8) vs. 2.5 (1.4–4.0)	Correlations between CpG sites, bisulphite batch, GA, maternal age, maternal education, parity, fetal sex, maternal BMI, folic acid, supplement use, smoking, preeclampsia
Qian (2016), China [34]	SGA:39 (41.0) AGA:49 (55.1)	Birth weight (birth)	*H19* (12 CpGs) & *MEST* (11 CpGs) using mass spectrometry-based method	Cord blood	Birth	Higher methylation levels in *H19* in SGA vs. AGA: Site 7.8: *p* = 0.03 Site 9: *p* = 0.02 Site 17.18: *p* = 0.05 No significant difference at other sites	
Hoyo (2014), US [35]	NEST, 496 (49.7)	Birth weight,(Birth 85% GAD >37w)	DMRs in *MEG3*,*NNAT*,*PEG10*/*SGCE*,*MEG3*-IG,*PLAGL1*,*PEG3*,*PEG1/MEST*,*H19*, *IGF2* using pyrosequencing	Cord blood	Birth (85% GAD >37w)	β (SE) for associations between DMR and birth weight (g). Referents are infants with methylation levels in the fourth quartile: *MEG3*:-10.92 (4.12), *p* = 0.008 *NNAT*: -7.57 (4.04), *p* = 0.06 *PEG10*/*SGCE*: 18.11 (5.77), *p* = 0.002 *MEG3*-IG: 0.42 (9.13), *p* = 0.96 *PLAGL1*: 12.33 (3.78), *p* = 0.001 *PEG3*: 1.36 (8.97), *p* = 0.88 *PEG1/MEST*: -5.60 (5.68), *p* = 0.33 *H19*: 20.25 (6.76), *p* = 0.003 *IGF2*: 6.10 (5.71), *p* = 0.29	Maternal race, sex, cigarette smoking, GAD, GA at blood draw, physical activity, pre-pregnancy BMI and delivery route
Hoyo (2012), US [36]	NEST, 300 (50.3)	Birth weight,(Birth, 85% GAD >37w)	DMR in *IGF2*, *H19* using pyrosequencing	Cord blood	Birth (85% GAD >37w)	DMR methylation fraction % (SD): *IGF2* Birth weight ≤2500g vs. >2500g: 48.6(9.4) vs. 48.3(7.5), *p* = 0.88 *H19* Birth weight ≤2500g vs. >2500g: 59.8(6.5) vs. 61.5 (8.0), *p* = 0.19	
Liu (2012), UK [37]	NEST, 508 (45)	Birth weight(Birth, GAD ≥37w)	DMRs in *IGF2*, *PEG10*, *PLAGL1* using pyrosequencing	Cord blood	Birth (GAD >37w)	% methylation difference in DMRs between NBW (2500-4500g), LBW (<2500g) & HBW (>4500g) *IGF2* DMR: 1.6% lower methylation among LBW vs. NBW *p* = 0.06 (female infants 2.3%, *p* = 0.03, black mothers 2.0%, *p* = 0.08) *PLAGL1* DMR: 5.9% higher methylation among HBW vs NBW, *p* = 0.02 *PEG10* DMR: 3.4% higher methylation among HBW compared with NBW, *p* = 0.06	
Soubry (2011), US [38]	NEST, 436 (47.5)	Birth weight (Birth)	*IGF2* DMR (3 CpGs) and *H19* DMR (4 CpGs) using pyrosequencing	Cord blood	Birth	Mean%(SD) *IGF2* methylation n = 356 < *2500g*: 47.57 (8.24) ≥*2500g*: 47.43 (6.67), Δ(*p*):-0.41 (0.91) Mean%(SD) *H19* methylation n = 411 < *2500g*: 58.96 (5.32) ≥*2500g*: 60.30 (7.58) Δ(*p*):+1.34 (0.15)	
Su (2016), China [39]	115 (NR)	Birth weight (Birth, all full term)	*IGF2* DMR (5 CpGs) using mass-spectrometry based method	Cord blood	Birth, all full term	Linear mixed model of *IGF2* methylation on birth weight accounting for correlations between CpG sites; Coef(*p*): *CpG1*:0.06(0.65) *CpG2*: -0.20(0.07) *CpG6*: -0.17(0.22) *CpG10*: -0.22(0.05), *p*<0.05 *CpG12*: -0.22(0.04), *p*<0.05 *GpG13*: -0.09(0.49) Linear mixed model of *H19* methylation on birth weight accounting for correlations between CpG sites; Coef(*p*): *CpG4*: 0.04(0.73) *CpG14*: -0.09(0.41) *CpG14-16*: 0.89(0.03), *p*<0.05 *CpG19-20*:0.27(0.07) *CpG23*: -0.23 (0.02), *p*<0.05 *CpG29*: -0.63 (0.09)	
Vidal (2013), US [40]	NEST 397 (51)	Birth weight (Birth)	*MEG3* & *PLAGL1* DMR, using pyrosequencing	Cord blood	Birth	β(SE) for DNA methylation at the *PLAGL1* & *MEG3* with a 10-g increase in birth weight: *MEG3*: -4.7(5.85), *p* = 0.42 *PLAGL1*: 10.47(5.22), *p* = 0.04	Infant sex, race, maternal education, maternal smoking, folic acid intake, GA at delivery
Zhang (2015), China [41]	SGA:60 (~40.7) AGA:60 (~40.7) LGA:30 (~40.7)	Birth weight (Birth)	*H19* and *IGF2* DMR using pyrosequencing	Blood	Birth	The methylation level of *H19* DMR was significantly higher in the SGA (*p* = 0.04) and LGA (*p* = 0.03) compared to AGA group	
Ghosh (2015), US [42]	LBW:57(NR) HBW:57(NR)	Birth weight(Birth)	96 CpG sites in genes found previously to be related to birth weight, growth and metabolism [23] using Infinium Human Methylation27k BeadChip	Cord blood	Birth	LBW infants had greater number (mean = 14) of disrupted CpGs/outliers than HBW children (mean = 5) (fishers exact test, *p* = 0.05)	
Azzi (2014), France [43]	EDEN,254 (NR)	Birth weight, Birth length, (Birth,GAD 39.5±1.5w)	*ZAC1* DMR methylation using allele-specific methylation multiplex real-time quantitative PCR	Cord blood	Birth (GAD 39.5±1.5w)	Spearman’s rank partial correlation coefficients for early life variables and *ZAC1* DMR methylation index: *Birth weight z-score*: 0.08 *p* = 0.23 *Birth length z-score*: 0.04 *p* = 0.51	Centre, child's sex, GA
Burris (2015), Mexico [44]	PROGRESS, 531 (45)	Birth weight (GAD38.8±1.8w)	*AHRR* gene promotor (3 CpGs) using pyrosequencing	Cord blood	Birth (GAD38.8±1.8w)	Average difference (95%CI) in *AHRR* DNA %methylation, across 3 CpG sites. Bonferroni adjustment *p* = 0.008: *Birth-weight-for-GA (per SD)*: -0.97 (-1.26, -0.85), *p*<0.0001	Maternal age, maternal BMI, maternal education, parity, smoke exposure, sex
Haworth (2013), UK [45]	129 (55)	Birth weight(Median GAD 39.4(IQR:39.0–40.3w))	Selection of sites based on [25]: *GSTM5* (2 CpGs), *HMOX2* (1 CpG), *ALOX12* (5 CpGs), *APOB* (7 CpGs), *AQP8* (1 CpG), *MAP2K3* (1 CpG), *AMN* (1 CpG) using pyrosequencing	Cord blood	Birth (Median GAD 39.4(IQR:39.0–40.3w))	Association between methylation % and BWP, *p* *GSTM5* site 1: 0.18 *GSTM5* site 2: 0.25 *HMOX2* site 1: 0.65 *ALOX12* site 1: 0.55 *ALOX12* site 2: 0.81 *ALOX12* site 3: 0.93 *ALOX12* site 4: 0.11 *ALOX12* site 5: 0.56 *APOB* site 1: 0.88 *APOB* site 2: 0.90 *APOB* site 3: 0.99 *APOB* site 4: 0.79 *APOB* site 5: 0.48 *APOB* site 6: 0.037, correlation r = -0.185 *APOB* site 7: 0.14 *AQP8* site 1: 0.71 *MAP2K3* site 1: 0.16 *AMN* site 1: 0.46 Associations between % methylation and proportion with low BWP (<50% vs. >50%) OR (95%CI) *GSTM5*: 0.33 (0.14–0.77), *p* = 0.01 *MAP2K3*: 0.24(0.01–0.83), *p* = 0.02 *APOB*: 2.56(1.14–5.76), *p* = 0.02 No significant associations for methylation in other genes (data not shown)	
Mulligan (2012), Democratic Republic of Congo [46]	25 (NR)	Birth weight (17% full term)	*NR3CI* (39 CpGs) using PCR	Cord blood	Birth (17% full term)	First PC of % methylation of 39 CpG sites explained 16.15% of variance & correlated with birth weight r = -0.45, *p* = 0.02	
Pan (2015), Singapore [47]	GUSTO, 991 (41)	Birth weight, Birth length, Body composition (Birth, GAD 38.9±1w)	*HIF3A* (3CpGs) using Infinium Human Methylation450 BeadChip	Cord blood	Birth (GAD 38.9±1w)	Association between methylation in cg27146050; cg16672562; cg22891070 and anthropometric outcomes. Coef i.e. % change in outcome for 10% increase in methylation (95% CI), *p* *Birth weight(g)*: 3.61 (0.68–6.63), *p* = 0.015; 3.34(1.4–5.3), *p* = 0.0007; 2.05(0.32–3.82), *p =* 0.20 *Birth length (cm)*: 0.60(-0.29–1.5), *p =* 0.19; 0.46(-0.13–1.05), *p =* 0.13; 0.35(-0.18–0.88), *p =* 0.20 *BMI at birth (g/cm*^*3*^*)*: 2.38(0.23–4.58), *p* = 0.03; 2.4(0.97–3.84), *p* = 0.00096; 1.35(0.07–2.64), *p* = 0.039 *Subscapular skinfold (mm)*: -0.77(-5.97–4.72), *p* = 0.78; 5.44(1.76–9.25), *p* = 0.0035; 3.27(0–6.64), *p* = 0.050 *Triceps skinfold (mm)*: *-*1.28(-6.42–4.14), *p* = 0.64; 0.75(-2.75–4.38), *p* = 0.68; 0.25(-2.90–3.51), *p* = 0.88 *Subscapular*:*triceps*: 0.50(-3.85–5.04), *p* = 0.83; 4.67(1.66–7.77), *p* = 0.0022; 3.02(0.34–5.75), *p* = 0.027	Child sex, ethnicity, cell type proportions and interactions between ethnicity and cell type proportions
Lesseur (2013), US [48]	Rhode Island Child Health Study, 58(~49)	Birth weight (Birth, GAD 39±1.1w)	*LEP* promotor using pyrosequencing	Cord blood	Birth (GAD 39 ±1.1w)	β coef (SE), *p* of *LEP* as dependent variable: AGA(reference) vs. LGA: 0.47(0.53), *p* = 0.31 AGA(reference) vs. SGA: 1.78(0.60), *p* = 4.6x10^-3^	Maternal blood LEP, pre-pregnancy BMI, race, tobacco use during pregnancy, hypertension during pregnancy, delivery method, maternal age, rs2167270 genotype, infant sex
**Childhood height/weight**							
Almén (2012), Greece [49]	Greek Healthy Growth Study, Normal weight: 24 (100) Obese: 23 (100)	Body size, (normal weight: 10.6± 0.5y & 10.5±0.4y for FTO A/T respectively)Obese: (11.1±0.9y & 10.7±0.5y for FTO A/T respectively)	Infinium Human Methylation27 BeadChip	Blood	Normal weight: 10.6± 0.5y & 10.5±0.4y for FTO A/T respectively Obese: 11.1±0.9y & 10.7±0.5y for FTO A/T respectively	Differentially methylated genes between obese and normal weight children: Average methylation (beta), % methylation change in obese relative to average methylation, *p* adjusted for multiple comparisons (CpG site NR): *CERCAM*: 4.9, -13.7%, *p* = 0.007 *DPYD*: 2.8, -16.4%, *p* = 0.008 *IL12A*: 2.8, 15.5%, *p* = 0.008 *ZNF35*: 22.2, -5.5%, *p* = 0.008 *ZNF362*: 5.2, -8.2%, *p* = 0.008 *TSC22D2*:6.4, 7.4%, *p* = 0.008 *CBX6*: 3.1, -16.7%, *p* = 0.008 *FOXF1*:4.7, -13.3%, *p* = 0.008 *PSMD7*: 7.5, -7.7%, *p* = 0.012 *H1FX*: 4.1, 10.2%, *p* = 0.02 *PRRC2C*: 4.1, -8.4%, *p* = 0.02 *MSI1*: 23.8, -3.8%, *p* = 0.02 *COL4A1*: 9.9, 8.6%, *p* = 0.02 *NBPF3*: 5.8, -8.4%, *p* = 0.02 *USP5*: 4.4, -10.4%, *p* = 0.03 *PLOD2*: 30.8, -5.4%, *p* = 0.03 *TLE3*: 5.5, -6.9%, *p* = 0.03 *RPS24*: 5.8, -10.0%, *p* = 0.04 *DVL3*: 4.4, 8.5%, *p* = 0.05 *POLD3*: 6.1, -8.8%, *p* = 0.05	
Perng (2012), Columbia [50]	BSCC,568(53.7)	Birth weight, Body size (5-12y)	LINE-1 using pyrosequencing	Blood	(5-12y)	LINE-1 methylation mean(SD) & birth weight (g), *All p*_*trend*_ *=* 0.90: <2,500: n = 44, 80.71(0.77) 2,500–2,999: n = 110, 80.28(0.67) 3,000–3,499: n = 128, 80.26(0.66) >3,500: n = 147, 80.22(0.62) *Males p*_*trend*_ = 0.72: <2,500: n = 20, 80.20(0.61) 2,500–2,999: n = 41, 80.49(0.73) 3,000–3,499: n = 57, 80.41(0.57) >3,500: n = 73, 80.30(0.64) *Females p*_*trend*_ = 0.87: <2,500: n = 24, 80.14(0.90) 2,500–2,999: n = 69, 80.16(0.60) 3,000–3,499: n = 71, 80.14(0.70) >3,500: n = 74, 80.13(0.59) LINE-1 methylation mean(SD) & height-for-age z-score *All p*_*trend*_ = 0.41: <-2.0: n = 55, 80.27(0.69) -2.0 -<-1.0: n = 176, 80.29(0.62) -1.0-<1.0: n = 299, 80.22 (0.69) ≥1.0: n = 20, 80.24 (0.33) *Males p*_*trend*_ = 0.75: <-2.0: n = 22, 80.27(0.64) -2.0 -<-1.0: n = 90, 80.40(0.57) -1.0-<1.0: n = 133, 80.37 (0.70) ≥1.0: n = 6, 80.34 (0.31) *Females p*_*trend*_ = 0.25: <-2.0: n = 33, 80.27(0.73) -2.0 -<-1.0: n = 86, 80.17(0.65) -1.0-<1.0: n = 166, 80.11 (0.65) ≥1.0: n = 14, 80.20 (0.34) LINE-1 methylation mean(SD) & BMI-for-age z-score *All p*_*trend*_ = 0.79: <-2.0: n = 10, 80.41(0.51) -2.0 -<-1.0: n = 63, 80.34(0.68) -1.0-<1.0: n = 371, 80.22 (0.64) 1.0-<2.0: n = 92, 80.31 (0.73) ≥2.0: n = 14, 80.28 (0.55) *Males p*_*trend*_ = 0.90: <-2.0: n = 5, 80.39(0.63) -2.0 -<-1.0: n = 30, 80.36(0.64) -1.0-<1.0: n = 160, 80.36 (0.64) 1.0-<2.0: n = 46, 80.42(0.70) ≥2.0: n = 10, 80.31 (0.57) *Females p*_*trend*_ = 0.42: <-2.0: n = 5, 80.43(0.43) -2.0 -<-1.0: n = 33, 80.32(0.72) -1.0-<1.0: n = 211, 80.11 (0.62) 1.0-<2.0: n = 46, 80.19 (0.75) ≥2.0: n = 4, 80.23 (0.58)	
Ouni (2016), NR [51]	ISS: 94(42) Normal height(control):119 (42)	Height (ISS 9±3y vs.control 10±3y)	*IGF1* promotor P1 (9 CpGs) & P2 (7 CpGs) using pyrosequencing	Blood	ISS: 9±3y Control: 10±3y	Mean methylation ± SD ISS children vs. controls, Bonferroni correction *p* (non-significant = 1) *IGF1* P1 promotor CG-1044: 88 ± 3 vs. 88 ± 3, *p* = 1 CG-960: 79 ± 2 vs. 79 ± 5, *p* = 1 CG-919: 90 ± 4 vs. 88 ± 6, *p* = 1 CG-631: 86 ± 2 vs. 86 ± 2, *p* = 1 CG-611: 93 ± 3 vs. 91 ± 3, *p* = 10^−4^ CG-491: 83 ± 4 vs. 83 ± 4, *p* = 1 CG-414: 12 ± 3 vs. 13 ± 5, *p* = 1 CG-308: 10 ± 4 vs. 10 ± 4, *p* = 1 CG-225: 8 ± 2 vs. 8 ± 2, *p* = 1 *IGF1* P2 promotor CG-232: 66 ± 7 vs. 63 ± 7, *p* = 0.005 CG-224: 74 ± 7 vs. 71 ± 7, *p* = 0.026 CG-218: 74 ± 7 vs. 70 ± 6, *p* = 0.008 CG-207: 45 ± 7 vs. 42 ± 7, *p* = 0.04 CG-137: 49 ± 4 vs. 46 ± 4, *p* = 9x10^-5^ CG-108: 61 ± 7 vs. 60 ± 6, *p* = 1 CG-77: 49 ± 6 vs. 47 ± 5, *p* = 1 CG+97: 17 ± 3 vs. 16 ± 3, *p* = 1	
Ouni (2015), NR [52]	Discovery cohort 110 (23) Replication 106 (41)	Height (9.7y boys; 9.6 girls)	*IGF1* promotor P1 (9 CpGs) & P2 (7 CpGs) using pyrosequencing	Blood	9.7y boys; 9.6y girls	Correlation between % methylation and child height Discovery cohort R,p(after Bonferroni correction); Replication cohort R,p(after Bonferroni correction); Total R; Total R,p(after Bonferroni correction) *IGF1* Promoter 1 CG-1044: 0.02, *p* = 1; 0.02, *p* = 1; 0.03, *p* = 1 CG-960: 0.05, *p* = 1; 0.06, *p* = 1; 0.06, *p* = 1 CG-919: 0.02, *p* = 1; -0.13, *p* = 1; 0.04, *p* = 1 CG-631: -0.04, *p* = 1; -0.04, *p* = 1; -0.09, *p* = 1 CG-611: -0.25, *p* = 0.1; -0.48, *p* = 2.2.10^−5^; -0.39, *p* = 4.10^−7^ CG-491: -0.06, *p* = 1; -0.31, *p* = 0.03; -0.10, *p* = 1 CG-414: 0.00, *p* = 1; -0.19, *p* = 0.4; 0.09, *p* = 1 CG-308: 0.02, *p* = 1; 0.02, *p* = 1; 0.00, *p* = 1 CG-225: 0.08, *p* = 1; 0.012, *p* = 1; -0.10, *p* = 1 *IGF1* P2 promotor CG-232: -0.08, *p* = 1; -0.28, *p* = 0.03; -0.21, *p* = 0.01 CG-224: -0.32, *p* = 10^−2^; -0.17, *p* = 0.7; -0.24, *p* = 0.003 CG-218: -0.36, *p* = 10^−3^; -0.30, *p* = 0.02; -0.33, *p* = 1.2.10^−5^ CG-207: -0.35, *p* = 2.10^−3^; -0.12, *p* = 1; -0.24, *p* = 4.10^−3^ CG-137: -0.30, *p* = 10^−2^; -0.40, *p* = 1.9.10^−4^; -0.36 *p* = 4.10^−7^ CG-108: -0.25, *p* = 0.10; -0.19, *p* = 0.5; -0.23 *p* = 0.12 Average (from -108 to -232): -0.31, *p* = 0.007; -0.27, *p* = 0.004; -0.3 *p* = 7.10^−5^ CG-77: -0.09, *p* = 1; -0.04, *p* = 1; -0.04 *p* = 1 CG+97: -0.08, *p* = 1; -0.03, *p* = 1; 0.07 *p* = 1	Age, sex
Hernandez-Valero (2013), US [53]	75 (40)	Body size,(8.2+-1.5y)	*H19* DMR (CpG4, SNP rs10732516)	Blood	8.2±1.5y	Association of CpG4 methylation status of H19 DMR (yes vs. no) with body size OR (95% CI): Girls: *Overweight vs*. *lean*: 0.39 (0.08, 2.02), *p* = 0.26 Boys: *Overweight vs*. *lean*: 3.14 (0.77, 12.89), *p* = 0.11 Overall: *Overweight vs*. *lean*: 1.27 (0.46, 3.54), *p* = 0.65	Maternal BMI, residence (urban vs. rural), sex
Gardner (2015), US [54]	64 (59.37)	Body size(5-6y)	Promoter regions of *FTO*, *MAOA*, *SH2B1*, *LEPR*, *DNMT3B*, *BDNF* and *CCKAR*using methylation-sensitive restriction enzyme digestion and qRTPCR	Saliva	(5-6y)	Mean(SD) BMI percentile according to *DNMT3B* methylation (based on percentile splits) *Lower tertile*: 86.24(17.87), *p* = 0.05 *Upper tertile* 72.89(23.78) (data from other genes not presented)	
**PROSPECTIVE**							
**Body size at birth**							
Agha (2016), US [55]	Project Viva, 476 (birth), 235 (7-10y) (48)	Birth weight,(Birth, all GAD >34w)	Infinium Human Methylation450 BeadChip	Cord blood &blood	Birth & (7-10y)	Adjusted difference (95% CI) in % cord blood methylation for 1 unit increase in birth weight for-GA z-score. FDR q <0.05: cg26663636(gene:*NOS1AP*): −0.39(−0.52, −0.25),*p* = 4.31x10^−09^ cg18181229(gene:*PBX1*): 1.86 (1.16, 2.56), *p* = 1.80x10^-07^ cg06750897(gene:*PBX1*): 1.93 (1.22, 2.64), *p* = 1.83x10^-07^ cg00222472(gene:*PBX1*): 1.78 (1.12, 2.45), *p* = 2.03x10^-07^ cg20682146(gene:*PBX1*): 1.54 (0.91, 2.17), *p* = 1.60x10^-06^ cg05780177(gene:*DENND1B*): 0.24 (0.14, 0.35), *p* = 2.39x10^-06^ cg00325458(gene:*REL*): 0.1 (0.06, 0.14), *p* = 7.27x10^-07^ cg23483765(gene:*NIPAL4*): 0.22 (0.14, 0.31), *p =* 1.15 x10^-07^ cg24353833(gene:*NRM*): 0.42 (0.27, 0.57), *p* = 8.38x10^-08^ cg24641186(gene:*TFAP2B*): 0.46 (0.27, 0.64), *p* = 1.41x10^-06^ cg20392842(gene:*HLA-DMB*):−1.58(−2.24,−0.92),*p* = 2.54x10^-06^ cg09364590(gene:*TIAM2*):0.72 (0.42, 1.03), *p* = 3.66x10^-06^ cg21809331(gene:*RBM28*):0.22 (0.13, 0.31), *p* = 2.28x10^-06^ cg14731462(gene:*PTPRE*):−0.92 (−1.29, −0.55), *p* = 4.20x10^-07^ cg25953130(gene:*ARID5B*):−2.01 (−2.8, −1.22), *p* = 7.76x10^-07^ cg23890469(gene:*MMRN2*): 0.57 (0.34, 0.81), *p* = 1.55x10^-06^ cg25124943(gene:-): −0.92 (−1.31, −0.53), *p* = 2.34x10^-06^ cg11606444(gene:*SORL1*): 0.68 (0.39, 0.98), *p* = 4.28x10^-06^ cg01345517(gene:*DERA*): 0.15 (0.09, 0.21), *p* = 1.55x10^-06^ cg06648759(gene:-): −1.08 (−1.52, −0.63), *p* = 2.02x10^-06^ cg14276580gene:-): −0.94 (−1.33, −0.54), *p* = 2.24x10^-06^ cg20549688(gene:*GTF2A2*): 0.21 (0.13, 0.29), *p* = 3.20x10^-07^ cg21842999(gene:*SHF*): 0.51 (0.3, 0.72), *p* = 3.20x10^-06^ cg09476997(gene:*SLC9A3R2*):1.81 (1.06, 2.57), *p* = 2.20x10^-06^ cg27283514(gene:-): 1.51 (0.86, 2.16), *p* = 3.34x10^-06^ cg19914554(gene:*CD7*): 0.73 (0.47, 0.98), *p* = 2.2x10^-08^ cg20186396(gene:*CD7*): 0.73 (0.45, 1.02), *p* = 3.26x10^-07^ cg14909906(gene:*KDSR*): 0.17 (0.1, 0.25), *p* = 2.37x10^-06^ cg23882285(gene:*ROCK1*): 0.12 (0.07, 0.17), *p* = 3.37x10^-06^ cg23026246(gene:*SPTBN4*): 0.13 (0.08, 0.19), *p* = 2.23x10^-06^ cg23877608(gene:*CCDC114*): 0.17 (0.1, 0.24), *p* = 2.35x10^-06^ cg23344780(gene:*EMP3*): −0.58 (−0.83, −0.33), *p* = 4.10x10^-06^ cg04803921(gene:*HM13*): 0.13 (0.08, 0.19), *p* = 1.68x10^-06^ cg08422803(gene:*ITGB2*): 1.05 (0.69, 1.41), *p* = 8.04x10^-09^ Of these 34 CpG sites, difference (95% CI) in % peripheral blood methylation at 7–10 years for 1 unit increase in birth weight for GA z-score. At FDR q value <0.05: cg26663636(gene: *NOS1AP*): Similar magnitude to cord blood cg18181229(gene: *PBX1*): Similar magnitude to cord blood cg00222472(gene: *PBX1*): Similar magnitude to cord blood cg20682146(gene: *PBX1*): 1.3 (0.5, 2.1)	Maternal age, race, education, smoking, parity, delivery mode, pre-pregnancy BMI, gestational diabetes, newborn sex, cord blood cell composition, childhood age and adult cell composition for prospective analyses
Broholm (2016), Denmark [56]	LBW: 13 (0) Control:13 (0)	Birth weight (Birth)	Infinium Human Methylation450 BeadChip	Adipose derived stem cells	LBW: 22.4±1.7y Control: 23.2 ± 1.6y	No significant difference in % methylation between LBW and control for individual CpG sites at FDR q<0.05. Top 20 CpGs (gene sites): cg14459772(gene:NA): *p* = 3.85×10^−7^ cg20170028(gene:*SHANK2*): p = 1.35×10^−6^ cg09528449(gene:*LY6H*): *p =* 2.31×10^−6^ cg13188409(gene:*SNX14*): *p* = 3.65×10^−6^ cg18530716(gene:*SLC16A11*): *p* = 3.65×10^−6^ cg20667124(gene:*NCLN*): *p* = 3.65×10^−6^) cg23032316(gene:*AAAS*): *p* = 3.65×10^−6^ cg26937500(gene:*CARD11*):*p* = 3.65×10^−6^ cg03726147(gene:*DMAP1*): *p* = 5.77×10^−6^ cg10143883(gene:*CEACAM19*): *p* = 5.77×10^−6^ cg10503854(gene:*EIF4A3*): *p* = 5.77×10^−6^ cg13598865(gene:*DYNLT1*): *p* = 5.77×10^−6^ cg00516515(gene:*CSK*): *p* = 8.65×10^−6^ cg01956781(gene:*FAM120B*): *p* = 8.65×10^−6^ cg04594598(gene:NA):*p* = 8.65×10^−6^ cg05759166(gene:NA):*p* = 8.65×10^−6^ cg22641201(gene:*BICC1*): *p* = 8.65×10^−6^ cg22777162(gene:*ACOX3*): *p* = 8.65×10^−6^ cg03947203(gene:*C2CD2L*): *p* = 1.29×10^−5^ cg04632980(gene:*TRIM11*): *p* = 1.29×10^−5^	
Simpkin (2015), UK [57]	ARIES: 1,018 (~51) Replication: WMHP, CANDLE & MoBa, (NR)	Birth weight, (Birth)	ARIES & MoBa: Infinium Human Methylation450 BeadChip WMHP & CANDLE: Infinium Human Methylation27BeadChip	Cord blood & blood	Birth, 7.5y, 17.1y	ARIES EWAS for birth weight, *p* <1.03x10^-7^ *Cord blood methylation*:23 probes in 14 genes (10 positive associations) *Blood at 7y/17y*: No strong evidence for birth weight and methylation Replication: Probes(gene), *p* with negative association between birth weight and methylation in ARIES and MoBa: cg20076442(NA): 6.01x10^-9^ cg25953130(ARID5B): 8.23x10^-9^ Probes(gene), *p* with negative association between birth weight and methylation in ARIES only: cg04521626(*PLD2*): 8.01x10^-11^ cg14097568(*NA*): 1.06x10^-9^ cg17133774(*CHD5*): 2.17x10^-9^ cg00654448(*NA*): 4.83x10^-9^ cg00442282(*RARA*): 7.77x10^-9^ cg13696490(*LOC201651*): 8.87x10^-9^ cg12044213(*CCHCR1*): 1.83x10^-8^ cg08817867(NA): 2.29x10^-8^ cg00382138(*CFI*) 2.50x10^-8^ cg06870470(*DOCK6*): 3.39x10^-8^ cg25557739(*NA*): 6.73x10^-8^ Probes(gene), *p* with positive association between birth weight and methylation in ARIES only: cg24324628(*NHSL1*): 2.62X10^-10^ cg15783940(NFIX): 1.33X10^-9^ cg14597739(*LTA*): 2.63X10^-9^ cg22962123(*HOXA3*): 3.12X10^-9^ cg05851442(*HOXA3*): 4.55X10^-9^ cg23387597(*ITPRIP*): 1.15X10^-8^ cg24973755(*MAEA*): 1.75X10^-8^ cg16219283(*LTA*): 1.94X10^-8^ cg25799241(*NA*): 6.46X10^-8^ cg06658067(*NA*): 7.05X10^-8^ Longitudinal analysis at identified probes Results suggest faster rates of change in methylation during childhood in children with low birth weight. No strong evidence for ages 7 to 17y	GA, parity, maternal age, maternal smoking, child sex, delivery method, cell type composition
Simpkin (2015), UK & Denmark [58]	AIRES 1018 (51) GOYA 981 (NR)	Birth weight, Body size (Birth & 7y)	Infinium Human Methylation450 BeadChip to estimate Horvath epigenetic age	Cord blood & blood	Birth, 7.5y, 17.1y	ARIES *Correlations between early life variable and age acceleration*: Birth weight (kg) & AA at birth: r = 0.01, *p* = 0.73 Birth weight (kg) & AA at 7 years: r = 0.08, *p* = 0.01 Birth weight (kg) & AA at 17 years: r = -0.07, *p* = 0.04 *Longitudinal analysis of AA* Birthweight (kg) & average AA during childhood and adolescence: r = 0.0003 (-0.001, 0.002), *p* = 0.72 Birthweight (kg) & changes with AA during childhood and adolescence: r = -0.00004 (-0.00017, 0.00009), *p* = 0.58 GOYA Birth weight (kg) was positively associated with newborn AA in GOYA (0.04y per kg, 95% CI 0.02, 0.07, *p* = 0.002)	Cell-type composition
Rerkasem (2015), Thailand [59]	249 (NR)	Birth weight, birth length (Birth)	COBRA LINE-1 & Alu	Blood	20y	% Total Alu/LINE-1 methylation, absence vs. presence mean(SD), *p* FDR *SGA*: *Alu*: 33.32(7.67) vs. 33.91(7.72), *p* = 0.14 *LINE-1*: 79.24(6.23) vs.80.21(5.96), *p* = 0.45 % UU methylation, mean(SD), *p* FDR *SGA*: *Alu*:40.50(8.78) vs. 44.61(9.36), p = 0.07 *LINE-1*: 9.27(6.22) vs. 7.31(4.99), *p* = 0.099 % MM methylation, mean(SD), *p* FDR *SGA*: *Alu*: 13.14(11.0) vs. 12.44(8.31), p = 0.70 *LINE-1*: 48.95(13.14) vs. 49.53(12.42), p = 0.77 % UM methylation, mean(SD), p FDR *SGA*: *Alu*: 25.56(7.89) vs. 24.06(6.73), p = 0.37 *LINE-1*: 18.67(13.63) vs. 21.26(13.25), p = 0.37 % MU methylation, mean(SD), *p* FDR *SGA*: *Alu*: 20.80 (6.89) vs. 18.90(5.54) p = 0.14 *LINE-1*: 23.11(21.90) vs. 21.90(9.02), p = 0.50 Correlation r(*p*)between % methylation and birth weight Total Alu: -0.10(0.22) Alu_UU:0.12(0.22) Alu_MM:-0.06(0.40) Alu_UM:0.03(0.67) Alu_MU:-0.11(0.22) Total LINE-1:0.13(0.22) LINE-1_MM:0.08(0.33) LINE-1_UU:-0.17(0.08) LINE-1_MU:-0.11(0.22) LINE-1_UM:0.07(0.35) Correlation r(*p*)between % methylation and birth length Total Alu: -0.01(0.51) Alu_UU:0.09(0.51) Alu_MM:-0.09(0.51) Alu_UM:0.05(0.65) Alu_MU:-0.07(0.62) Total LINE-1:0.05(0.65) LINE-1_MM:0.01(0.88) LINE-1_UU:-0.10(0.51) LINE-1_MU:-0.02(0.85) LINE-1_UM:0.04(0.65)	
Terry (2008), US [60]	85 (100)	Birth weight, Birth length (Birth)	Global DNA methylation using [3H]-methyl acceptance assay	Blood	(38-48y)	Unadjusted differences log DPM/μg(95% CI) for association between DNA methylation by variables(higher values indicated less DNA methylation *Birth weight kg*: -0.04 (-0.27, 0.18) *Birth length cm*: -0.05 (-0.10, -0.002) Multivariate linear regression DPM/μg(95% CI) for association between DNA methylation by variables *Birth weight kg*: 0.28 (-0.11, 0.67) *Birth length cm*: -0.11 (-0.19, -0.03)	Smoke exposure, adult BMI, SEP, parity
Drake (2012), UK [61]	The Motherwell Cohort, 34(64)	Birth weight, Birth length Ponderal index; (Birth, GAD: 272.5±5.5d)	Promotor region of *HSD2*, exon 1(C) and 1(F) of *GR*, *IGF2* DMRs using pyrosequencing	Blood	40±0.12y	Pearson correlation coefficients of mean methylation with birth weight *HSD2 Region1*: 0.49, *p*<0.05; partial correlation 0.48, *p* = 0.01 *HSD2 Region 2*: 0.05, *p*>0.05 *H19 ICR*: -0.02, *p*>0.05 *GR Exon 1F*: 0.08, *p*>0.05 *GR Exon 1C*: 0.13, *p*>0.05 Pearson correlation coefficients of mean methylation with birth length *H19 ICR*: -0.36, *p*<0.05 Pearson correlation coefficients of mean methylation ponderal index *HSD2 Region1*: 0.23, *p*>0.05 *HSD2 Region 2*: 0.20, *p*>0.05	GAD, parity, sex, maternal antenatal BMI
Hernandez-Valero (2013), US [53]	75 (40)	Birth weight, (Birth)	*H19* DMR (CpG4, SNP rs10732516)	Blood	8.2±1.5y	Association of CpG4 methylation status of H19 DMR (yes vs. no) with body size OR (95% CI): Girls: *Birth weight above vs*. *birthweight below median (7*.*1lbs)*: 0.07 (0.007, 0.74), *p* = 0.03 Boys: *Birth weight above vs*. *birth weight below median (7*.*1lbs)*: 0.58 (0.14, 3.34), *p* = 0.44 Overall: *Birthweight above vs*. *birthweight below median (7*.*1lbs)*: 0.32 (0.11, 0.94), *p* = 0.04	Maternal BMI, residence (urban vs. rural), sex
Steegers-Theunissen (2009), The Netherlands [62]	HAVEN study control 120 (~58)	Birth weight (Birth, GAD ~39w)	*IGF2* DMR (5 CpGs) using mass-spectrometry based method	Blood	17m	*IGF2* DMR %(SE) of mean change in relative methylation *Birth weight*: -1.7(0.8), *p* = 0.03	Periconceptional folic acid use, GA
Wehkalampi (2013), Finland [63]	The Helsinki Study of VLBW Adults: VLBW:158(58) Controls:161 (60)	Birth weight	*IGF2* (IGF2AS & IGF205) DMR using Sequenom EpiTYPER	Blood	(18-27y)	Mean (SD) methylation % at IGF2 VLBW (≤1,500g) vs. control *IGG2AS*: CpG3:55.6(0.04) vs. 57.4(0.05) CpG4: 60.4(0.07) vs. 60.9(0.06) CpG67: 39.9(0.04) vs. 40.7(0.04) CpG8: 51.8(0.05) vs. 52.8(0.04) *IGF2_05*: CpG12: 68.5(0.05) vs. 68.4(0.05) CpG34 67.4(0.5) vs. 67.1(0.05) CpG6: 50.6(0.04) vs. 50.7(0.04) CpG7: 56.3(0.05) vs. 56.4(0.04) CpG8: 55.4(0.04) vs. 55.3(0.04) CpG91011: 53.1(0.05) vs. 52.7(0.05) Differences (95% CI) in methylation between VLBW vs. controls *IGG2AS*: CpG3: -0.017(-0.028, -0.005), *p* = 0.004 CpG4: -0.010(-0.026, 0.007), *p* = 0.25 CpG67: -0.008(-0.017, 0.0001), *p* = 0.10 CpG8: -0.008(-0.020, 0.004), *p* = 0.18 *IGF2_05*: CpG12: 0.004(-0.008, 0.017), *p* = 0.51 CpG34: 0.005(-0.008, 0.018), *p* = 0.44 CpG6: -0.002(-0.012, 0.009), *p* = 0.78 CpG7: 0.001 (-0.011, 0.012), *p* = 0.93 CpG8: 0.003(-0.008, 0.013), *p* = 0.61 CpG91011: 0.008(-0.004, 0.021), *p* = 0.17	Plate, sex, age, height, BMI, mother’s smoking during pregnancy, mother’s age, father’s age, mother’s BMI before pregnancy, highest education of either parent
Obermann-Borst (2013), the Netherlands [64]	120 (42)	Birth weight (Birth)	*LEP* using mass-spectrometry based method	Blood	17± 2.5m	% Absolute methylation change (SE); % relative methylation change (SE) from linear mixed model *Model 1—each variable in model separately* Birth weight (SD): -1.2 (0.4); -5.0(1.7), *p* = 0.005 Growth rate (SD): 0.0 (0.4); 0.0 (0.3), *p* = 0.99 *Model 2—adjusted for all variables p <0*.*1 in Model 1* Birth weight: -0.6(0.5); -2.5(2.1), *p* = 0.16	Model 1 Correlation between individual CpG dinucleotides, bisulfite batch, GA Model 2 Correlation between individual CpG dinucleotides, bisulfite batch, GA, education, smoking, breastfeeding, sex, serum leptin, BMI
Tao (2013), US [65]	639 (100) breast cancer cases	Birth weight,(Birth)	*E-cadherin*, *p16 and RAR-β2*, using PCR	Breast tumor tissue	57.5±11.3y	OR (95%CI) for methylation *E-cadherin* *Premenopausal group*: Birth weight ≤2.5kg: 2.79(1.15,6.82) 2.6–3.9kg: ref >3.9kg: 1.69(0.70,4.05) *Postmenopausal group*: Birth weight ≤2.5kg: 0.77(0.38,1.54) 2.6–3.9kg: ref >3.9kg: 0.86(0.42,1.73) *P16* *Premenopausal group*: Birth weight ≤2.5kg: 0.70(0.27,1.85) 2.6–3.9kg: ref >3.9kg: 0.79(0.33,1.88) *Postmenopausal group*: Birth weight ≤2.5kg: 0.66(0.34,1.26) 2.6–3.9kg: ref >3.9kg: 0.68(0.35,1.34) *RAR-β2*, *Premenopausal group*: Birth weight ≤2.5kg: 1.00(0.39,2.57) 2.6–3.9kg: ref >3.9kg: 1.61(0.72,3.60) *Postmenopausal group*: Birth weight ≤2.5kg: 1.03(0.57,1.85) 2.6–3.9kg: ref>3.9kg: 1.20(0.65, 2.22)	Age, education, race, oestrogen receptor status
Rangel (2014), Brazil [66]	115 (47)	Birth weight (Birth)	*ACE* (3 CpGs) using pyrosequencing	Blood	(6-12y)	Methylation levels (% average over 3 CpGs): LBW (≤2.5kg): 5.4±0.28% NBW (≥3kg): 6.8±0.19% LBW children had lower methylation at CpG1 (*p* = 0.001) and CpG3 (*p* = 0.009). No significant difference at CpG2 (*p* = 0.14) Adjusted model, *p<0*.*001* LBW (≤2.5kg): 5.1(4.7, 5.8) NBW (≥3kg): 6.8(6.4, 7.2)	Premature status, sex, age, BMI, family history of CVD
**Childhood body size and growth**							
Simpkin (2015), UK & Denmark [58]	AIRES 1018 (51) GOYA 981 (NR)	Birth weight, body size (Birth & 7y)	Infinium Human Methylation450 BeadChip to estimate Horvath epigenetic age	Cord blood & blood	Birth, 7.5y, 17.1y	ARIES *Correlations between early life variable and age acceleration*: Height at 7y & AA 7 years: r = 0.06, p = 0.06 Height at 7y & AA at 17 years: r = 0.06, p = 0.07 BMI at 7y & AA 7 years r = :0.037, *p* = 0.25 BMI at 7y & AA at 17 years: r = 0.005, *p* = 0.88	Cell-type composition
Rerkasem (2015), Thailand [59]	249 (NR)	Growth (birth, 3, 6, 9, 12 months)	COBRA LINE-1 & Alu	Blood	20y	% Total Alu/LINE-1 methylation, absence vs. presence mean(SD), *p* FDR *Catch up growth*: *Alu*: 33.66(6.99) vs. 39.61(7.22), *p*<0.00001 *LINE-1*: 79.87(5.52) vs. 79.74(8.95), *p* = 0.94 % UU methylation, mean(SD), *p* FDR *Catch up growth*: *Alu*: 44.85(8.76) vs.37.39(8.71), *p*<0.00001 *LINE-1*: 7.86 (5.40) vs. 8.35(6.21), *p* = 0.78 % MM methylation, mean(SD), *p* FDR *Catch up growth*: *Alu*: 12.16(9.07) vs.16.60(8.58), p = 0.233 *LINE-1*: 49.09(12.20) vs. 50.49(18.26), p = 0.78 % UM methylation, mean(SD), p FDR *Catch up growth*: *Alu*: 23.75(6.95) vs.26.04(7.11), p = 0.19 *LINE-1*: 20.78(14.19) vs. 20.98(15.46), *p* = 0.94 % MU methylation, mean(SD), *p* FDR *Catch up growth*: *Alu*: 19.23(6.06) vs.19.97(6.55), p = 0.78 *LINE-1*: 22.28(9.35) vs. 20.19(10.10), p = 0.45	
Groom (2012), UK [67]	Cohort 1: Newcastle Preterm Birth Growth Study; Cohort 2: ALSPAC (see results for n’s)	Postnatal growth: (cohort 1: 10–16 wk; cohort 2: birth-8 weeks) Fat mass: (cohort 1: median 12y, IQR: 3, cohort 2: 10y, IQR = 0.3)	*TACSTD2* (7 CpGs) using pyrosequencing	Cord blood, blood	Cohort 1: median 12y, IQR:3 Cohort 2: birth & 7y	Postnatal growth and *TACSTD2* methylation Mean methylation % Slow growers vs. rapid growers, Spearman rank correlation, *p*: *Cohort 1 n = 94*: 73.05(53.15–76.79) vs. 76.18(57.04–78.99), 0.23, *p* = 0.027 *Cohort 2 (methylation at 7y) n = 161*: 68.45(53.95–73.55), 0.10, *p* = 0.29 Childhood fat mass and *TACSTD2* methylation Spearman rank correlation, *p* *Cohort 1* (median age for fat mass 12, IQR = 3), n = 91: -0.22, *p* = 0.037 Cohort 2 (median age for fat mass 10y, IQR = 0.3): *Cord blood methylation*, *n = 131*: 0.20, *p* = 0.04 *Methylation at 7y*, *n = 144*: 0.17, *p* = 0.068	
**TWIN STUDIES**							
Chen (2016), Denmark [68]	DTR, 150 MZ twin pairs(48)	Birth weight(Birth)	Infinium Human Methylation450 BeadChip	Blood	Median 57y (30–74)	No genome-wide significant DMR at FDR <0.2 for qualitative discordance (large or small). One DMR for Δbw% with FDR = 0.128 covers 11 CpGs on chromosome 1 (hg19 chr1:75,198,211–75,199,117) where two genes, *CRYZ* and *TYW3* are located cg17719053 cg10128416 cg21906852 cg09502221 cg07399417 cg26855724 cg02709834 cg26690034 cg26752657 cg21535942 cg00121533	Age, sex, batch effects
Tan (2014), Denmark [69]	DTR, 150 MZ twin pairs (28 pairs extremely discordant) (48)	Birth weight (Birth)	Infinium Human Methylation450 BeadChip	Blood	median 57y (30–74)	No genome-wide significant CpG associated with qualitative (large or small) or quantitative (Δbw%) birth weight discordance at FDR <0.05. Age-dependent intra-pair differential methylation in extremely discordant twins (Δbw% >25%) at 5% FDR: cg26856578(gene:NA):0.003, *p* = 3.42×10^−8^ cg15122603(gene:NA):-0.002, *p* = 1.25×10^−7^ cg16636641(gene:*ZCCHC2*):-0.018, *p* = 2.05×10^−7^	WBC count estimates, age, sex, batch effects
Tsai (2015), UK [70]	TwinsUK (discovery): 71 MZ pairs (100). DTR old (replication): 27 (48.1) DTR young (replication): 29 (51.7) NTR (replication): 89 (74.2)	Birth weight (Birth)	Infinium Human Methylation450 BeadChip	Blood	TwinsUK 55.4±9.84y DRT (old): 64.1±4.7y DRT (young): 33.8 ±1.6y NTR: 34.2±12.1y	Spearman’s rank correlation coefficient (95% CI) Discovery at 5% FDR: cg12562232(gene:*IGF1R*): 0.603, (0.430, 0.719), *p* = 2.62×10^−8^ Replication: DTR old: 0.263, (-0.130, 0.585), *p* = 0.186 DTR young: -0.027, (-0.390, 0.343), *p* = 0.888 NTR: 0.161, (-0.049, 0.357, *p* = 0.132) Meta-analysis: All twins: 0.282, (-0.037, 0.550), *p* = 0.041 Old twins: 0.474, (0.099, 0.731), *p* = 0.008). Next top-ranked signals in discovery (FDR = 0.57): cg12049992(gene:*FAM38B*): -0.519, *p* = 3.49×10^−6^ cg12508856(gene:*KIF13B*): -0.510, *p* = 5.52×10^−6^ cg12391576(gene:*HLA-DPA1*): 0.508, *p* = 6.07×10^−6^ cg26313699(gene:*OR1G1*): -0.507, *p* = 6.29×10^−6^	Sex, age, cell type composition, smoking status, alcohol consumption, methylation plate, position on the plate, family, and zygosity
Casey (2017), Canada [71]	Quebec Newborn Twin Study, 52 pairs of MZ twins (58)	Birth weight,(Birth)	Infinium Human Methylation450 BeadChip	Saliva	15.7±0.3y (15.3–16.7)	No one gene locus was significantly differentially methylated in birth weight discordant MZ twin pairs after correcting for multiple testing. CpGs (gene) below *p*<0.0004 cg06313433(gene:*FLNG*), *p* = 2.38×10^−6^ cg11967457(gene:*LEFTY2*), *p* = 2.89×10^−5^ cg18755581(gene:*TATDN3*), *p* = 8.95×10^−5^ cg09608383(gene:*FAM189A1*), *p* = 9.89×10^−5^ cg18790856 (gene:NA), *p* = 1.14×10^−5^ cg17316316(gene:NA), *p* = 1.27×10^−5^	Cell type composition, sex, family
Baird (2011), NR** [72]	10 MZ twin pairs	Birth weight (Birth)	Infinium Human Methylation27 BeadChip	Peripheral blood mononuclear cells	Adult (NR)	No one gene locus was significantly differentially methylated in all birth weight discordant MZ twin pairs. 21 loci were statistically significant differentially methylated in at least 4 of the 10 twin pairs, including *INSR*	
Gordon (2012), Australia [73]	22 MZ and 12 DZ twin pairs (50)	Birth weight, (GAD 36.2±1.8wk (32–38))	Infinium Human Methylation27 BeadChip	Cord blood, umbilical vascular cells, placenta	GAD 36.2±1.8wk (32–38)	Genewise linear models with twin-pair as a factor and birth weight as covariate: CpGs, (gene), B, adjusted *p* (FDR p<0.1) Cord Blood MZ: cg23366752 (gene:*DNAJA4*): 0.73, *p* = 0.27 cg26136776 (gene:*KLF1*): -0.15, *p* = 0.49 cg22290566 (gene:*LMAN1L*): -0.23, *p* = 0. 49 cg02921257 (gene:*CMYA1*): -0.27, *p* = 0.49 cg02989940 (gene: *ERAF*): -0.54, *p* = 0.49 cg11653858 (gene: SLC13A2): -0.75, *p* = 0.49 cg01564343 (gene:*TREML1*): -0.76, *p* = 0.49 cg11692021 (gene: *GNB3*): -0.89, *p* = 0.49cg03569412 (gene: *MBD3*): -0.89, *p* = 0.49 cg16204289 (gene: *FLJ13391*): -0.913, *p* = 0.49 Cord Blood DZ: cg13750354 (gene:*OCIAD1*): 4.78, *p* = 0.009 cg07469792 (gene:*RASSF8*): 4.13, *p* = 0.014 cg23807559 (gene:*COG1*): 3.73, *p* = 0.018 cg20166532 (gene:*EDG1*): 2.97, *p* = 0.038 cg13501117 (gene:*WDR8*): 2.92, *p* = 0.038 cg16192916 (gene:*PSME4*): 2.44, *p* = 0.062 cg09419670 (gene:*PSMD5*): 2.01, *p* = 0.097 cg00400263 (gene:*C20orf177*): 1.87, *p* = 0.10 cg24329794 (gene:*GRSF1*): 1.61, *p* = 0.13 cg10488141 (gene:*SUFU*): 1.50, *p* = 0.13 Umbilical cells MZ: cg02813863 (gene:*APOLD1*): -4.07, *p* = 0.07 cg26196700 (gene *SORD*): -4.19, *p* = 0.999 cg11546621 (gene: *PTGDS*): -4.20, *p* = 0.999 cg26244225 (gene: *APOLD1*): -4.23, *p* = 0.999 cg01366419 (gene: *WBSCR17*): -4.23, *p* = 0.999 cg10673984 (gene: *PPM1D*): -4.25, *p* = 0.999 cg03962522 (gene:*SLC5A1*): -4.25, *p* = 0.999 cg00303548 (gene:*HS6ST3*): -4.25, *p* = 0.999 cg12888961 (gene:*PCTK2*): -4.27, *p* = 0.999 cg08832227 (gene:*KCNA1*): -4.27, *p* = 0.999 Umbilical cells DZ: cg00766729 (gene: *LOC147808*): 1.57, *p* = 0.14 cg01962826 (gene: *GRM4*): 1.31, *p* = 0.14 cg18320336 (gene: *STEAP1*): 1.20, *p* = 0.14 cg04007936 (gene: *CARHSP1*): 1.07, *p* = 0.14 cg22238923 (gene: *DOK1*): 1.004, *p* = 0.14 cg20909686 (gene: *OVOL1*): 0.996, *p* = 0.14 cg02196730 (gene: *MTHFS*): 0.51, *p* = 0.28 cg20803370 (gene: *PPOX*): 0.28, *p* = 0.37 cg15437432 (gene: *TMED9*): 0.14, *p* = 0.41 cg08256260 (gene: *KCTD13*): 0.03, *p* = 0.44 Placenta MZ cg17554194 (gene:*HLA-B*): -3.48, *p* = 0.34 cg23508052 (gene:*SCD*): -3.55, *p* = 0.56 cg15869642 (gene:*CBLN1*): -3.57, *p* = 0.56 cg14614211 (gene:*IRXL1*): -3.59, *p* = 0.57 cg23337754 (gene:*CRABP1*): -3.62, *p* = 0.68 cg23442323 (gene:*CD109*): -3.66, *p* = 0.68 cg26976437 (gene:*LY6K*): -3.66, *p* = 0.68 cg12259537 (gene:*ZNF606*): -3.66, *p* = 0.68 cg24134767 (gene:*HTR3A*): -3.67, *p* = 0.68 cg14601284 (gene:*PLXDC1*): -3.67, *p* = 0.68 Placenta DZ cg04189838 (gene: *CYP2C19*): -3.39, *p* = 0.73 cg10430690 (gene: *KALRN*): -3.43, *p* = 0.73 cg02764897 (gene: *KRTAP13-1*): -3.48, *p* = 0.73 cg10918419 (gene: *C8orf55*): -3.49, *p* = 0.73 cg06324671 (gene: *KRTAP19-7*): -3.49, *p* = 0.73 cg13757640 (gene: *ARHGAP8*): -3.50, *p* = 0.73 cg12924408 (gene:*RABL4*): -3.51, *p* = 0.73 cg26610808 (gene:*BLOC1S2*): -3.51, *p* = 0.73 cg08088390 (gene:*DEFB125*): -3.51, *p* = 0.73 cg11530960 (gene:*DMRT2*): -3.52, *p* = 0.73	Batch effects
Córdova-Palomera (2014), Spain [74]	17 MZ twin pairs (47)	Birth weight,(Birth)	248 CpGs sites at t: *IGF2*, *IGF2BP1*, *IGF2BP2*, *IGF2BP3* measured using Infinium Human Methylation450 BeadChip	Blood	37.8±11.2y (22–56)	Inter-individual association (i.e. not birth weight discordance) between *IGF2BP1* methylation (mean methylation of cg07075026 & cg20566754) and birth weight: β = 83.3 x10^-3^ *p* = 0.033. Each kg increase in birth weight corresponding to approximately 8.33% rise in methylation fraction. Other results with birth weight NR	Sex, age, IQ, and GA
Souren (2013), Belgium [75]	EFPTS, 17 MZ monochorionic twin pairs (100)	Birth weight (GAD 37.9±2.4w (34–42))	Infinium Human Methylation450 BeadChip & LINE-1 & HERVK using methylation-dependent primer extension assays (SIRPH)	Saliva	34.4± 7.1y (22–45)	3,153 CpGs differentially methylated between heavy and light co-twins (*p*<0.01), of which 45 show sensible absolute mean methylation differences (β-value difference >0.05) Validation analysis of 8 selected BW-MVPs mean difference(SD) in heavy vs. light twins: cg14123607(*APBA1*): 0.07(0.05), *p* = 0.0008 cg12170649(*APPL2*): -0.06(0.05), *p*<0.0001 cg26404226(*NA*): -0.05(0.04), *p*<0.0001 cg15487251(*IGF2BP2*): -0.05(0.05), *p* = 0.002 cg10362113(*PAPOLA*): 0.06(0.07), *p* = 0.008 cg02409150(*PHKG2*): -0.06(0.05), *p*<0.0001 cg15049370(*PPARGC1B*): -0.07(0.07), *p* = 0.002 cg22768222(*RUNX2*): 0.06 (0.07), *p* = 0.008 Differences remain in the range of technical variation, arguing against a reproducible biological effect. Analysis of methylation in repetitive elements showed no significant intra-pair differences.	Cell composition
Mill (2006), UK [76]	TEDS, 12 MZ twin pairs (50)	Birth weight, (Birth)	*COMT* using pyrosequencing	Buccal	5y	Average methylation difference (%) between birth weight discordant pairs: CpG1: 10.3 CpG2: 16.1 Average: 13.19	

*Studies spanning more than one exposure may appear twice in the table;

**Abstract;

AA: Age acceleration; Δbw%: Percentage of Birth Weight Difference; AGA: Average for Gestational Age; ALSPAC: Avon Longitudinal Study of Parents and Children; ARIES: Accessible Resource for Integrated Epigenomic Studies; BMI: Body Mass Index; BSCC: Bogota School Children Cohort; BWP: Birth Weight Percentile; BW-MVP: Birth Weight-Associated Methylation Variable Positions; CANDLE: Conditions Affecting Neurocognitive Development and Learning in Early Childhood Study; CCCEH: The Northern Manhattan Mothers and Newborns Study of the Columbia Center for Children’s Environmental Health; CI: Confidence Interval;COBRA: Combined Bisulfite Restriction Analysis; CVD: Cardiovascular Disease; DMR: Differentially Methylated Regions; DPM: Disintegrations Per Minute; DTR: Danish Twin Registry; DZ: Dizygotic twins; EFPTS: East Flanders Prospective Twin Survey; EWAS: Epigenome Wide Association Study;FDR: False Discovery Rate; FT: Full Term; GAD: Gestational age at delivery; GUSTO: Growing up in Singapore towards Healthy Outcomes; HBW: High Birth Weight; IQR: Interquartile Range; ISS: Idiopathic Short Stature; LBW: Low birth weight; LGA: large for gestational age; LUMA: Luminometric Methylation Assay; M: Months; MoBa: Norwegian Mother and Child Cohort; MZ: Monozygotic twin; NBW: Normal Birth Weight; NEST: Newborn Epigenetics Study; NGT: Normal Glucose Tolerance; NR: Not Reported; NTR: Netherlands Twin Register; OR: Odds Ratio; PAH: Princess Anne Hospital Study; PC: Principle Component; PROGRESS: Programming Research in Obesity, Growth Environment and Social Stress; qRTPCR: Reverse Transcriptase Plymerase Chain Reaction; SD: Standard Deviation; SE: Standard Error; SGA: Small for Gestational Age: SEP: Socioeconomic Position; SVA: Surrogate Variable Analysis; SWS: Southampton Women’s Study; TEDS: Twins Early Development Study; THREE: Baltimore Tracking Health Related to Environmental Exposures Study; VLBW; Very Low Birth Weight; VPT: Very Preterm; W: Week; WMHP: Women’s Mental Health Program; Y: Years;

**Table 2:** Nutrition in early life and epigenetics. *(Organised by, *exposure*, *DNA methylation (epigenome wide*, *global methylation*, *imprinted genes*, *other genes)*.

First author (year), country	Cohort, N (% female)	Early life variable(mean age ± SD (age range)	DNA methylation	Tissue	Mean age at epigenetic measure ± SD (age range)	Main result	Confounders
**Maternal dietary intake / nutritional biomarker**							
Joubert (2016), Norway & The Netherlands [85]	MoBA: 1275 (NR) Generation R: 713 (NR)	Plasma folate	Infinium Human Methylation450 BeadChip	Cord blood	Birth	443 FDR-significant CpGs were differentially methylated in cord blood in relation to maternal folate. 48 CpGs met Bonferroni threshold (p<1.19x10^-7^). Selected loci from meta-analysis, Coef(SE), p: cg15908975(*GRM8*):-0.012(0.002),6.76x10^-7^ cg18574254(*GRM8*):-0.011(0.002),3.27x10^-9^ cg22591480(*SLC16A12*):-0.008(0.002),1.34x10^-5^ cg14920044(*SLC16A12*):-0.011(0.003), 4.31x10^-6^ cg24829292(*OPCML*):0.010(0.002),6.60x10^-6^ cg 22629528(*OPCML*):0.019(0.005),2.91x10^-5^ cg 26283170(*OPCML*):0.009(0.002), 1.30x10^-5^ cg 24804179(*PRPH*):-0.007(0.002), 8.05x10^-6^ cg 05775627(*PRPH*):-0.007(0.002),1.01x10^-5^ cg 16010628(*PRPH*):-0.005(0.001), 1.73x10^-5^ cg 05635274(*PRSS21*):0.009(0.002),4.77x10^-6^ cg 02296564(*PRSS21*):0.011(0.003),6.21x10^-6^ cg 22730830(*PRSS21*):0.013(0.003),3.99x10^-6^ cg 01232511(*PRSS21*):0.014(0.003), 1.23x10^-5^ cg 10612259(*LHX1*):-0.011(0.002), 9.10x10^-8^ cg 011965477(*LHX1*):-0.002(0.001), 2.09x10^-5^ cg 11775595(*APC2*):-0.015(0.003), 1.64x10^-7^ cg 14907738(*APC2*):-0.006(0.001), 8.57x10^-6^ cg 27150718(*APC2*):-0.009(0.002),5.81x10^-7^ cg 03165176(*APC2*):-0.012(0.003),1.44x10^-5^ cg 14559388(*APC2*):-0.003(0.001),4.98x10^-6^ cg 04624885(*APC2*):-0.010(0.002), 1.56x10^-5^ cg 19870717(*APC2*):-0.009(0.002),4.64x10^-9^ cg 16613938(*APC2*):-0.016(0.003),3.05x10^-8^ cg 23291200(*APC2*):-0.010(0.002),1.72x10^-9^ cg 13793157(*KLK4*):-0.009(0.002), 4.00x^10-5^ cg10078829(*KLK4*):-0.007(0.002), 1.84x10^-5^	Maternal age, education, smoking during pregnancy, parity, batch effects
Boeke (2012), US [86]	Project Viva, Periconceptional intake: 516 Second trimester intake: 484 (47.7)	FFQ for B-vitamins (32 ± 5.1y)	LINE-1 using pyrosequencing	Cord blood	Birth	0–4 weeks gestation, β = %5MC difference in LINE-1 methylation for increment in 1 SD in nutrient *Methyl donor(Cumulative Index)*: β = -0.02 (-0.04, 0.01), *p* = 0.17 *maternal vitamin B12 (*μ*g/d)*: β = 0.01 (-0.06,0.08), *p* = 0.70 *maternal betaine (mg/d)*: β = -0.04 (-0.11,0.03), *p* = 0.24 *maternal choline (mg/d)*: β = -0.02 (-0.08,0.04), *p* = 0.45 *maternal folate (*μ*g/d)e*: β = -0.03 (-0.10,0.03), *p* = 0.32 Second trimester, β = %5MC difference in LINE-1 methylation for increment in 1 SD for nutrients *maternal vitamin B12*: β = -0.02 (-0.09,0.06), *p* = 0.64 *maternal betaine*: β = -0.02 (-0.10,0.05), *p* = 0.50 *maternal choline*:β = -0.004 (-0.07,0.06), *p* = 0.98 *maternal folate*: β = -0.02 (-0.08,0.05), *p* = 0.61	Other methyl donors, child's sex, mother's age, race, smoking, pregnancy, weight gain, education, cadmium intake
Pauwels (2017), Belgium [87]	MANOE, 115(47.8)	FFQ for methyl donor intake & folic acid supplementation (31±3.6y)	Global DNA methylation using mass-spectrometry method & *DNMT1*, *LEP*, *RXRA*, *IGF2* DMR using PCR	Cord blood	Birth (GAD 39.6±0.9w)	Before pregnancy (n = 24) β(95%CI), *p*: *LEP*: Betaine: -0.13(-3.45, 3.19), p = 0.94 Choline: 1.48(-1.48, 4,45), p = 0.31 Folate: -0.33(-2.75, 2.09), p = 0.78 Methionine: 0.427 (0.01, 0.85), p = 0.048 *DNMT1*: Betaine:0.675(0.04, 1,31), p = 0.039 Choline:0.13(-0.52,0.78), p = 0.68 Folate:0.21(-0.3, 0.72), p = 0.40 Methionine: 0.04(-0.06, 0.14), p = 0.37 Second trimester (n = 89) β(95%CI), *p*: *LEP*: Betaine:-0.575(-1.16, 0.01), p = 0.05 Choline:-0.47(-0.95, 0.02), p = 0.058 Folate:-0.507(-0.89, -0.13), p = 0.009 Methionine: -0.06(-0.14, 0.02), p = 0.15 *DNMT1*: Betaine:-0.25(-0.58, 0.09), p = 0.15 Choline: -0.301(-0.57, -0.03), p = 0.03 Folate:-0.226(-0.45, -0.01), p = 0.045 Methionine: -0.04(-0.08, 0.009), p = 0.12 Third trimester (n = 89) β(95%CI), *p*: *RXRA*: Betaine: 0.35(-1.24, 1.94), p = 0.66 Choline:-0.935(-2.08, 0.21), p = 0.11 Folate:-1.001(-1.96, -0.04), p = 0.041 Methionine: -0.15(-0.35, 0.06), p = 0.16 *DNMT1*: Betaine:0.97(0.36, 3,67), p = 0.96 Choline:0.291(0.1. 0.84), p = 0.022 Folate:0.48(0.22, 1.06), p = 0.07 Methionine: 0.87 (0.74, 1.04), p = 0.12 Folic acid supplementation *LEP CpG1 methylation* > 6 months before conception vs. 3–6 months before conception: 34.6 ± 6.3% vs. 30.1 ± 3.6%, p = 0.011 *LEP CpG3 methylation* > 6 months before conception vs no supplement before conception: 16.2 ± 4.4% vs. 13.9 ± 3%, p = 0.036 *RXRA mean methylation* supplements during entire pregnancy vs. stopping in second trimester: 12.3 ± 1.9% vs. 11.1 ± 2%, p = 0.008	Maternal age, maternal BMI, maternal smoking before and during each trimester of pregnancy, gestational weight gain
Fryer (2009), UK [88]	24 (58.3)	Folic acid supplementation during pregnancy (29.4±7y)	LINE-1 methylation using pyrosequencing	Cord blood	Birth	Correlation with LINE-1 methylation: *Maternal folic acid intake*: β = 0.31, *p* = 0.15 *Prescribed folic acid dose during pregnancy*: β = 0.36, *p* = 0.31	Sex, GA maternal age, parity, and BMI and cord serum folate, plasma homocysteine
Haggarty (2013), UK [81]	913 (46)	FFQ for folate intake, folic acid supplementation, RBC folate (30.5 (95%CI: 30.2–30.9y)	*IGF2* (4 CpGs), *PEG3* (7 CpGs), *SNRPN* (15q11, 4 CpGs) *LINE*-*1* (4 CpGs) using pyrosequencing	Cord blood	Birth (GAD 3.95 (95%CI: 39.4, 39.6w))	LINE-1 methylation: *maternal folate intake (100ug/d)*:β = 0.002 (-0.20,0.20), *p* = 0.98 *maternal folate supplement use*, *yes/no (periconceptional)*:β = 0.05 (-0.25,0.35), *p* = 0.74 *maternal folate supplement use*, *yes/no (first 12 weeks gestation)*: β = 0.16 (-0.23,0.55), *p* = 0.42 *maternal folate supplement use*, *yes/no (after 12 weeks)*: β = -0.34 (-0.64,-0.04), *p* = 0.03 *maternal RBC folate*, *100 nmol/L*:β = -0.13 (-0.20,-0.05), *p* = 0.001 PEG-3 methylation: *maternal folate intake (100ug/d)*:β = 0.002 (-0.20,0.2), *p* = 0.44 *maternal folate supplement use*, *yes/no (periconceptional)*:β = -0.02 (-0.40,0.37), *p* = 0.94 *maternal folate supplement use*, *yes/no (first 12 weeks gestation)*: β = -0.47 (-0.86,-0.08), *p* = 0.02 *maternal folate supplement use*, *yes/no (after 12 weeks)*: *maternal RBC folate*, *100 nmol/L*: β = -0.02 (-0.10,0.06), *p* = 0.60 SNRPN methylation: *maternal folate intake (100ug/d)*: β = 0.07 (-0.33,0.46), *p* = 0.74 maternal folate supplement use, yes/no (periconceptional): β = -0.22 (-0.36,0.81), *p* = 0.46 *maternal folate supplement use*, *yes/no (first 12 weeks gestation)*: β = 0.39 (-0.37,1.15), *p* = 0.32 *maternal folate supplement use*, *yes/no (after 12 weeks)*: β = -0.01 (-0.60,0.58), *p* = 0.97 *maternal RBC folate*, *100 nmol/L*:β = 0.02 (-0.12,0.15), *p* = 0.82 IGF2 methylation: *maternal folate intake (100ug/d)*: β = 0.23 (-0.21,0.67), *p* = 0.32 *maternal folate supplement use*, yes/no (periconceptional): β = 0.31 (-0.35,0.96), *p* = 0.36 *maternal folate supplement use*, *yes/no (first 12 weeks gestation)*: β = -0.10 (-0.95,0.76), *p* = 0.83 *maternal folate supplement use*, *yes/no (after 12 weeks)*: β = 0.68 (0.02,1.35), *p* = 0.04 *maternal RBC folate*, *100 nmol/*L:β = 0.10 (-0.05,0.24), *p* = 0.18	
McKay (2012), UK [89]	The North Cumbria Community Genetics Project, Infant: 294 (48) Maternal: 121	Serum B12 (median 28.6y)	Global DNA methylation using LUMA & *IGF2*, *IGFBP3*, *ZNT5* using pyrosequencing	Cord blood	Birth	Global DNA methylation correlated inversely with maternal vitamin B12 concentrations: β = 0.0002(0.0001), *p =* 0.06. After adjustment: serum B12:β = 0.00007 (0.00007), *p* = 0.29	Sex, GA, infant *MTHFR* genotype
Hoyo (2011), US [90]	NEST 428 (50)	Folic acid supplement before (n = 428) and during pregnancy (n = 223) (29 ± 6.2y)	*IGF2* & *H19* DMR using pyrosequencing	Cord blood	Birth	Methylation % difference for folic acid supplement before pregnancy: *IGF2 methylation*: *Moderate vs*. *non-users*: 0.28, *p* = 0.76 *High (i*.*e*. *prescribed & over the counter*) *vs*. *non-users*: -1.15, p = 0.39 *H19 methylation*: *Moderate vs*. *non-users*:-1.96, *p* = 0.03 *High vs*. *non-users*: -2.76, p = 0.04 Methylation % difference for folic acid supplement during pregnancy: *IGF2 methylation*: *Moderate vs*. *non-users*:0.75, *p* = 0.59 *High vs*. *non-users*: 0.25, p = 0.93 *H19 methylation*: *Moderate vs*. *non-users*:-2.87, *p* = 0.02 *High vs*. *non-users*: -4.90, p = 0.05	Maternal education, race, mode of delivery, cigarette smoking, sex
Steegers-Theunissen (2009), The Netherlands [62]	HAVEN study controls 120(~58)	Folic acid supplementation during pregnancy 400 μg/day vs. no supplement	*IGF2* (5 CPGs) using mass-spectrometry based method	Blood	17 months	Mean (SE) of IGF2 methylation in childhood without maternal exposure to folic acid n = 34 vs. exposed n = 86: 0.474(0.007) vs. 0.495(0.004), *p* = 0.014 Adjusted analysis: mean difference in IGF2 methylation 4.5% (1.8) with maternal exposure to folic acid vs unexposed, *p* = 0.014	Maternal education
Loke (2013), Australia [91]	PETS 95 twin pairs (55 MZ & 40 DZ) (~50%)	Folate and macronutrient intake	*IGF2 and H19* DMRs using mass-spectrometry based method	HUVECs, (CBMCs and granulocytes); ectoderm (buccal epithelium) and extra embryonic ectoderm (placenta)	Birth (GAD median 37.0±1.94w)	Difference (*p*) in absolute percentage methylation in all tissues combined All Assays combined *Had folate*: 0.50(0.44) *Vitamin B12 (z-score)*: -0.23(0.24) *Homocysteine(z-score)*: 0.27(0.29) *Macronutrients (z-score*): 0.37(0.17) H19 promoter DMR *Had folate*: -1.70(0.024) *Vitamin B12 (z-score)*: -0.97(0.002) *Homocysteine(z-score)*: 0.10(0.75) *Macronutrients (z-score*): 0.80(0.049) IGF2/H19 ICR *Had folate*: 0.40(0.69) *Vitamin B12 (z-score)*: -0.23(0.54 *Homocysteine(z-score)*: 0.40(0.29) *Macronutrients (z-score*): 0.20(0.050) IGF2 DMR0 *Had folate*: 0.90(0.46) *Vitamin B12 (z-score)*:0.23(0.55) *Homocysteine(z-score)*: 0.37(0.30) *Macronutrients (z-score*): 0.43(0.27) IGF2 DMR2 *Had folate*: 2.90(0.035) *Vitamin B12 (z-score)*:0.27(0.63) *Homocysteine(z-score)*: 0.17(0.72) *Macronutrients (z-score*): 0.10(0.77) Differences in coefficients between cell types Had folate: HUVECs vs buccal -4.5%; p = 0.026; Vitamin B12 z-score: Granulocytes vs buccal (2.1%; p = 0.004). No other difference found	
Azzi (2014), France [43]	EDEN 254(NR)	FFQ for B-vitamins & supplementation (during pregnancy (29.8±4.4y))	*ZAC1* DMR using methylation-specific PCR	Cord blood	Birth (GA at birth 39.5±1.5)	Spearman’s rank partial correlation coefficients Prior to pregnancy: *Vitamin B2*: 0.14 *p* = 0.04 *Vitamin B3*: 0.04, *p* = 0.60 *Vitamin B6*: 0.04, p = 0.49 *Vitamin B9*: 0.02, p = 0.74 *Vitamin B12*: 0.11, p = 0.08 Last 3 months of pregnancy: *Vitamin B2*: 0.11 *p* = 0.09 *Vitamin B3*: 0.08, *p* = 0.22 *Vitamin B6*: 0.04, p = 0.5 *Vitamin B9*: 0.04, p = 0.56 *Vitamin B12*: 0.02, p = 0.79 No association with folic acid supplementation and/or the use of a combination of micronutrients either prior to or during pregnancy (estimates not provided)	
Obermann-Bors (2013), The Netherlands [64]	120 (50)	Folic acid supplementation	*LEP* using mass-spectrometry based method	Blood	17± 2.5m	Variable, % absolute methylation change (SE), p No folic acid: 0.1(0.8) p = 0.91	Batch, correlation between 7 CpGs,
Adkins (2010), NR**[92]	30 (NR)	Biomarkers on one carbon pathway	*~*15,000 loci (Details not specified)	NR	Birth	Phosphatidyl choline was significantly correlated with newborn DNA methylation at a subset of loci	
Ba (2011), China [93]	99 (48)	B-vitamin biomarker (27.8 ±5.3y)	*IGF2* promoters using methylation-specific PCR	Cord blood	Birth (96% GAD 37-41w)	Promoter P2: Mean change per SD of each characteristic (*p*): *Maternal blood serum folate*: 0.05 (0.47) *Maternal blood serum vitamin B12*: 0.09 (0.19) Promoter P3: Mean change per SD of each characteristic (*p*): *Maternal blood serum folate*: 0.049 (0.47) *Maternal blood serum vitamin B12*: -0.22 (0.001)	Mother’s age, maternal prepregnancy BMI, weight gain during pregnancy, mother’s highest education level, parity, supplementation intake during pregnancy, birth weight and birth length, sex, and GA
Hoyo (2014), US [35]	NEST 496 (49.7)	Erythrocyte folate (first trimester)	*IGF2*, *H19*, *DLK1*, *MEG3*, *PEG3*, *MEST*, *PEG10*, *SGCE*, *NNAT* using pyrosequencing	Cord blood	Birth	Erythrocyte folate quartiles β(SE): *MEG3 methylation*:β = -2.02 (0.58), *p* = 0.001 for Q4 vs Q1 *NNAT methylation*:β = -1.34 (0.73, *p* = 0.07 for Q3 vs Q1 *PEG10/SEGCE methylation*:β = -0.14 (0.33), *p* = 0.66 for Q4 vs Q1 *MEG3-IG methylation*:β = -0.68 (0.61), *p* = 0.27 for Q4 vs Q1 *PLAG1 methylation*:β = -1.01 (0.40), *p* = 0.01 for Q3 vs Q1 *PEG3 methylation*:β = 0.43 (0.22), *p* = 0.03 for Q2 vs Q1 *PEG3/MEST methylation*:β = 0.39 (0.44), *p* = 0.37 for Q4 vs Q1 *H19 methylation*:β = 0.09 (0.33), *p* = 0.78 for Q4 vs Q1 *IGR2 methylation*:β = -0.04 (0.43), *p* = 0.004 for Q2 vs Q1	Maternal race, sex, cigarette smoking, GAD, GA at blood draw, physical activity, pre-pregnancy BMI, and delivery route
McCullough (2016), US [94]	NEST 429 (50)	B-vitamin biomarkers (56% between 20-29y)	*H19 MEG3 SGCE/PEG10 PLAGL1 DMR* using pyrosequencing	Cord blood	Birth	*H19* methylation β(SE) *serum B12*:β = -0.41 (0.57), *p* = 0.48 for Q4 vs Q1 *serum pyridoxal phosphate*: β = -0.07 (0.63), *p* = 0.91 for Q4 vs Q1 *serum 4-pyridoxic acid*:β = -0.57 (0.61), *p* = 0.35 for Q4 vs Q1 *serum homocysteine*: β = 1.01 (0.59), *p* = 0.09 for Q2 vs Q1 *MEG3* methylation β(SE) *serum B12*:β = -0.93 (0.85), *p* = 0.27 for Q4 vs Q1 *serum pyridoxal phosphate*: β = 3.24 (0.89), *p*<0.01 for Q4 vs Q1 *serum 4-pyridoxic acid*:β = 1.62 (0.87), *p* = 0.06 for Q4 vs Q1 *serum homocysteine*: β = 1.60 (0.87), *p* = 0.07 for Q4 vs Q1 *SGCE/PEG10* methylation β(SE) *serum B12*:β = 0.47 (0.67), *p* = 0.48 for Q4 vs Q1 *serum pyridoxal phosphate*: β = -0.30 (0.81), *p* = 0.71 for Q4 vs Q1 *serum 4-pyridoxic acid*:β = 1.46 (0.74), *p* = 0.05 for Q2 vs Q1 *serum homocysteine*: β = 1.43 (0.77), *p* = 0.06 for Q2 vs Q1 *PLAG1* methylation β(SE): *serum B12*:β = 1.79 (0.96), *p* = 0.06 for Q4 vs Q1 *serum pyridoxal phosphate*:β = -0.11 (1.04), *p* = 0.91 for Q4 vs Q1 *serum 4-pyridoxic acid*:β = -0.15 (0.99), *p* = 0.88 for Q4 vs Q1 *serum homocysteine*: β = 1.77 (0.97), *p* = 0.07 for Q3 vs Q1	GAD, GA at blood draw, maternal race/ethnicity, maternal smoking and pre-pregnancy body mass index
Dominguez-Salas (2014), The Gambia [95]	Keneba Cohort 126 (43)	One-carbon metabolism biomarkers (18-45y)	Metastable epialleles: *BOLA3*, *LOC654433*, *EXD3*, *ZFVE28* using methylation-specific amplification microarray and pyrosequencing. *RBM46*, *PARD6G*, *ZNF678* using pyrosequencing	Blood lymphocytes (n = 126), Hair follicle (n = 87)	3.6 ±0.9m	Effect sizes are 1)standardised β coefficient for change in mean DNA methylation (combined MEs) per 1 SD of the predictor and 2) odds ratio per change in predictor: Peripheral blood lymphocyte: *serum folate nmol/* *l*: β = 0.02(-0.07,0.12), OR = 1.03 (0.90,1.17), *p* = 0.62 *serum vitamin B2 1/EGRAC*:β = 0.09 (0.00,0.19), OR = 1.19 (0.98,1.46), *p* = 0.05 *serum vitamin B12 pmol/l*: β = 0.03 (-0.07,0.14), OR = 1.04 (0.91,1.19), *p* = 0.54 *serum active vitamin B12 pmol/l*: β = -0.04 (-0.16,0.07), OR = 0.98 (0.87–1.11), *p* = 0.45 *serum choline umol/l*: β = -0.01 (-0.12,0.09), OR = 0.95 (0.80–1.12), *p* = 0.80 *serum betaine umol/l*: β = 0.05 (-0.10,0.20), OR = 1.03 (0.89–1.19), *p* = 0.49 *serum dimethyl glycine umol/*l:β = -0.06 (-0.16,0.04), OR = 0.95 (0.86,1.04), *p* = 0.21 *serum betaine/dimethyl glycine*:β = 0.08 (-0.02,0.17), OR = 1.05 (0.97,1.14), *p* = 0.11 *serum S-adenosylmethionine nmol/l*: β = -0.06 (-0.17,0.05), OR = 0.79 (0.58,1.08), *p* = 0.28 *serum S-adenosylhomocysteine nmol/*l: β = -0.09 (-0.18,0.01), OR = 0.88 (0.75,1.02), *p* = 0.07 *maternal serum S-adenosylmethionine/S-adenosylhomocysteine*: β = 0.06 (-0.03,0.15), OR = 1.08 (0.92,1.27), *p* = 0.18 *serum methionine umol/l*: β = 0.07 (-0.03,0.18), OR = 1.19 (0.90,1.56), *p* = 0.18 *serum homocysteine umol/l*: β = -0.14 (-0.23,-0.05), OR = 0.80 (0.68,0.93), *p* = 0.003 *maternal serum vitamin B6 nmol/l*: β = -0.16 (-0.27,-0.04), OR = 0.82 (0.71,0.94), *p* = 0.005 *serum cysteine umol/l*: β = -0.19 (-0.31,-0.07), OR = 0.45 (0.30,0.68), *p* = 0.002 Hair follicle: *serum folate nmol/l*: β = 0.01 (-0.11,0.13), OR = 1.00 (0.86,1.16), *p* = 0.81 *serum vitamin B2 1/EGRAC*:β = 0.11 (0.00,0.22), OR = 1.22 (0.97,1.53), *p* = 0.04 *serum vitamin B12 pmol/l*: β = 0.08 (-0.06,0.23), OR = 1.06 (0.88,1.26), *p* = 0.25 *serum active vitamin B12 pmol/l*:β = -0.03 (-0.18,0.13), OR = 1.00 (0.85,1.18), *p* = 0.75 *serum choline umol/l*: β = 0.01 (-0.13,0.14), OR = 0.96 (0.77,1.19), *p* = 0.91 *serum betaine umol/l*: β = 0.13 (-0.07,0.32), OR = 1.06 (0.88,1.28), *p* = 0.19 *serum dimethyl glycine umol/l*:β = -0.02 (-0.15,0.11), OR = 0.97 (0.86,1.09), *p* = 0.79 *serum betaine/dimethyl glycine*:β = 0.06 (-0.06,0.18), OR = 1.04 (0.94,1.15), *p* = 0.34 *serum S-adenosylmethionine nmol/l*: β = -0.05 (-0.19,0.09), OR = 0.85 (0.57,1.27), *p* = 0.48 *serum S-adenosylhomocysteine nmol/l*:β = -0.12 (-0.25,0.01), OR = 0.84 (0.69,1.03), *p* = 0.06 *serum S-adenosylmethionine/S-adenosylhomocysteine*: β = 0.09 (-0.03,0.22), OR = 1.15 (0.93,1.41), *p* = 0.13 *serum methionine umol/l*: β = 0.00 (-0.13,0.14), OR = 0.99 (0.70,1.38), *p* = 0.96 *serum homocysteine umol/l*: β = -0.15 (-0.27,-0.03), OR = 0.82 (0.67,1.00), *p* = 0.02 *maternal serum vitamin B6 nmol/l*: β = -0.12 (-0.26,0.02), OR = 0.86 (0.73,1.02), *p* = 0.08 *serum cysteine umol/l*: β = -0.20 (-0.36,-0.04), OR = 0.43 (0.25,0.72), *p* = 0.01	
Rerkasem (2015), Thailand [59]	249(NR)	24-hour food recall & FFQ in each trimester	*LINE-1* and *Alu* using COBRA	Blood	20y	% Total methylation, r, *p*(FDR) *Maternal Protein intake 1*^*st*^ *trim*: *Alu*: 0.18, p = 0.46 *LINE-1*: -0.11, p = 0.75 *Maternal Protein intake 2nd*^*t*^ *trim*: *Alu*: -0.08, p = 0.61 *LINE-1*: 0.08, p = 0.61 *Maternal Protein intake 3*^*rd*^ *trim*: *Alu*: 0.04, p = 0.78 *LINE-1*: 0.06, p = 0.78 *Maternal CHO intake 1*^*st*^ *trim*: *Alu*: 0.05, p = 0.81 *LINE-1*: -0.05, p = 0.82 *Maternal CHO intake 2*^*nd*^ *trim*: *Alu*: 0.01, p = 0.87 *LINE-1*: -0.05, p = 0.88 *Maternal CHO intake 3*^*rd*^ *trim*: *Alu*: 0.07, p = 0.74 *LINE-1*:0.06, p = 0.73 *Maternal fat intake 1*^*st*^ *trim*: *Alu*: -0.11, p = 0.64 *LINE-1*: -0.22, p = 0.46 *Maternal fat intake 2*^*nd*^ *trim*: *Alu*: -0.09, p = 0.87 *LINE-1*: -0.007, p = 0.98 *Maternal fat intake 3*^*rd*^ *trim*: *Alu*: -0.17, p = 0.09 *LINE-1*:0.006, p = 0.96 *Maternal energy intake 1*^*st*^ *trim*: *Alu*: 0.03, p = 0.82 *LINE-1*: -0.11, p = 0.54 *Maternal energy intake 2*^*nd*^ *trim*: *Alu*: -0.02, p = 0.92 *LINE-1*: -0.03, p = 0.91 *Maternal energy intake 3*^*rd*^ *trim*: *Alu*: -0.008, p = 0.92 *LINE-1*:0.05, p = 0.88	
Drake (2012), UK [61]	The Motherwell Cohort, 34(64)	FFQ (early ≤20w & late pregnancy >20w)	*HSD2* (promotor region), exon 1(C) and 1(F) of *GR* (exon 1(C) and 1(F)), *IGF2* DMRs using pyrosequencing	Blood	40 (0.12y)	Correlation of mean GR exon 1F methylation during late pregnancy Meat/w: r = 0.48, p = 0.009 Fish/w: r = 0.38, p = 0.048 Veg/w: r = 0.67, p<0.001 Bread/w: r = -0.49, p = 0.009 Potato/w: r = -0.39, p = 0.04 Methylation was increased at a specific CpG sites in *HSD2* with increased meat (r = 0·42, *p* = 0·03) and fish r = 0·40, *p* = 0·04) intake in late pregnancy. Other results not presented	Sex, BMI, birth weight.
Godfrey (2011), UK [96]	PAH 78 (NR)	FFQ (GA 15w)	*eNOS*, *SOD1*, *IL8*, *P13KCD*, *RXRA* using pyrosequencing	Cord blood	Birth	Higher methylation of *RXRA* but not of *eNOS* was associated with lower maternal CHO intake. Maternal fat and protein intake were not associated with *RXRA* methylation. No estimates for other nutrients/genes	
Simpkin (2015), UK [58]	AIRES 1018 (51)	Serum selenium & vitamin D	Infinium Human Methylation450 BeadChip to estimate Horvath epigenetic age	Cord blood & blood	Birth, 7.5y, 17.1y	Correlations between early life variable and age acceleration: Maternal selenium & AA at birth:-0.103, p = 0.06 Maternal selenium & AA 7 years: -0.137, p = 0.009 Maternal selenium & AA at 17 years: 0.01, p = 0.84 Maternal vitamin D & AA at birth:-0.05, p = 0.20 Maternal vitamin D & AA at 17 years: -0.002, p = 0.95 Maternal vitamin D & AA at 17 years: -0.009, p = 0.82	Cell-type composition
**Early life dietary intake / nutritional biomarker**							
Simpkin (2015), UK [58]	AIRES 1,018 (51)	Breastfeeding	Infinium Human Methylation450 BeadChip to estimate Horvath epigenetic age	Cord blood & blood	Birth, 7.5y, 17.1y	Correlations between early life variable and age acceleration: Breastfeeding & AA at birth: r = 0.035, p = 0.30 Breastfeeding & AA at 7 years: r = -0.010, p = 0.76 Breastfeeding & AA at 17 years: r = 0.026, p = 0.43	Cell-type composition
Rossnerova (2013), Czech Republic [97]	Asthmatics:100 (45). Controls:100(45)	Breastfeeding	Infinium Human Methylation27 BeadChip	Blood	11.6±2y	Breastfeeding was associated with overall DNA methylation, but no statistical test performed	
Obermann-Borst (2013), The Netherlands [64]	120 (50)	Breastfeeding	*LEP* using mass-spectrometry based method	Blood	17± 2.5m	% absolute methylation change (SE), p Duration breast feeding: -0.6 (0.2), *p* = 0.04	Batch, correlation between 7 CpGs, birth weight, growth rate, smoking, BMI, GA, sex folic acid
Tao (2013), US [65]	639 (100) breast cancer cases	Breastfeeding	*E-cadherin*, *p16 and RAR-β2*, using PCR	Breast tumour tissue	57.5y ±11.3	OR (95%CI) for methylation breastfed yes (ref) vs no *E-cadherin* *Premenopausal group*: 1.21(0.50,2.93) *Postmenopausal group*:1.06(0.64,1.77) *P16* *Premenopausal group*: 2.75(1.14,1.67) *Postmenopausal group*:0.79(0.49,1.26) *RAR-β2*, *Premenopausal group*: 1.18(0.53,2.62) *Postmenopausal group*: 1.30(0.83–2.04)	Age, education, race, oestrogen receptor status
Wijnands (2015), UK [98]	120 (41.7)	Breastfeeding & lipid biomarkers	*LEP* & *TNFα* using mass-spectrometry based method	Blood	17±2.5m	%Absolute methylation change (i.e. methylation change per SD change in biomarker (SE)) *TNFα* Total cholesterol: -1.0(0.5), p = 0.036. (Additional adjustment for HDL attenuated the results p = 0.07) Triglycerides: 0.1(0.5), p = 0.773 HDL-cholesterol:-1.2(0.5), p = 0.013. (Adjustment for maternal HDL slightly attenuated the association p = 0.08) LDL- cholesterol:-0.8(0.5), p = 1.00 %Absolute methylation change (β(SE)) *LEP* Total cholesterol:-0.6(0.3), p = 0.11 Triglycerides: 0.1 (0.4), p = 0.71 HDL-cholesterol:-3.4 n(1.5), p = 0.02. (Adjustment for maternal HDL slightly attenuated the association p = 0.041) LDL- cholesterol: -1.7 (1.5), p = 0.25 Bonferroni correction attenuated to nonsignificant estimates *TNFα* methylation was not associated with duration of breastfeeding. *LEP* methylation was significantly associated with duration of breastfeeding: -0.6 (95%CI -1.19, -0.01) per increment in breastfeeding duration category	Bisufite batch
Fryer (2011), UK [25]	12 (92)	Plasma homocysteine (birth)	Infinium Human Methylation27 BeadChip	Cord blood	Birth	Two clusters were identified following unsupervised hierarchical clustering to identify underlying methylation β-value across samples. Plasma homocysteine was lower (p = 0.038) in cluster B. There was no difference in serum folate (estimates not presented). 298 CpGs associated with plasma homocysteine (*p*<0.05)	
Fryer (2009), UK [88]	24 (58.3)	Plasma homocysteine & serum folate (birth)	LINE-1 methylation using pyrosequencing	Cord blood	Birth	Correlation with LINE-1 methylation: *Cord plasma homocysteine*: β = -0.69, *p* = 0.001 (p = 0.004 following adjustment) *Cord serum folate*: β = 0.21, *p* = 0.34	Sex, GA, maternal age, parity, BMI, serum folate, and maternal folic acid intake
McKay (2012), UK [89]	The North Cumbria Community Genetics Project 294 (48)	RBS folate & serum B12 (GA 39.5 ± 1.4w)	Global DNA methylation using LUMA & *IGF2*, *IGFBP3*, *ZNT5* using pyrosequencing	Cord blood	Birth	Methylation of the *IGFBP3* locus inversely correlated with infant vitamin B12 concentration (r = -0.16, p = 0.007)	Sex, GA, infant *MTHFR* genotype
Nafee (2009), UK** [31]	24(NR)	Homocysteine (birth)	LINE-1	Cord blood	Birth	LINE-1 methylation levels were inversely correlated with cord blood homocysteine (p = 0.01, r = -0.688)	
Perng (2012), Columbia [50]	BSCC 568(53.7)	Erythrocyte folate, plasma vitamin B12, vitamin A ferritin (an indicator of iron status), serum zinc concentrations (5-12y)	LINE-1 using pyrosequencing	Blood	(5-12y)	LINE-1 methylation β(95%CI) & Erythrocyte Folate (nmol/L), *All p*_*trend*_ = 0.51: Q1: n = 139, ref Q2: n = 139, -0.03(-0.18, 0.11) Q3: n = 139, 0.01(-0.14, 0.16) Q4: n = 139, 0.04(-0.11, 0.19) LINE-1 methylation β(95%CI) & plasma B12 (pmol/L), *All p*_*trend*_ *=* 0.51: Q1: n = 137, ref Q2: n = 136, -0.04(-0.19, 0.11) Q3: n = 134, 0.06(-0.22, 0.09) Q4: n = 136, -0.12(-0.28, 0.04) LINE-1 methylation β(95%CI) & serum zinc (umol/L), *All p*_*trend*_ = 0.60: Q1: n = 140, ref Q2: n = 142, 0.014(-0.14, 0.16) Q3: n = 141, 0.07(-0.08, 0.23) Q4: n = 141, 0.02(-0.14, 0.18) Adjusted: LINE-1 methylation β(95%CI) & plasma ferritin (ug/L), *All p*_*trend*_ = 0.22: Q1: n = 141, ref Q2: n = 139, -0.16(-0.31, -0.01) Q3: n = 143, -0.08(-0.24, 0.07) Q4: n = 141, -0.13(-0.28, 0.03) LINE-1 methylation β(95%CI) & plasma vitamin A (umol/L), *All p*_*trend* =_ 0.006: <0.700: ref 0.70–1.05: -0.07(-0.24, 0.10) ≥:1.050, -0.19(-0.36, -0.02)	Sex, vitamin A, CRP, maternal BMI, household socioeconomic position
Ba (2011), China [93]	99 (48)	B-vitamin biomarkers (96% GAD 37-41w)	*IGF2* 2 promoters using methylation-specific PCR	Cord blood	Birth (96% GAD 37-41w)	Promoter P2: Mean change per SD of each characteristic (*p*): *cord blood serum folate*: 0.18 (0.07) *cord blood serum vitamin B12*: -0.03 (0.75) Promoter P3: Mean change per SD of each characteristic (*p*): *cord blood serum folate*:-0.03 (0.77) *cord blood serum vitamin B12*: -0.04 (0.60)	Mother's age, maternal pregnancy BMI, weight gain during pregnancy, mother's highest education level, parity, supplementation intake during pregnancy, Newborn's birth weight and birth length, Newborn's sex and GA
Haggarty (2013), UK [81]	913 (46)	RBS folate (GAD: 39.5 (95%CI: 39.4, 39.6w))	*IGF2* (4 CpGs), *PEG3* (7 CpGs), *SNRPN* (15q11, 4 CpGs) *LINE*-*1* (4 CpGs) using pyrosequencing	Cord blood	Birth	LINE-1 methylation: *cord RBC folate 100 nmol/L*: β = -0.08 (-0.12,-0.03), *p* = 0.001 PEG-3 methylation: *cord RBC folate 100 nmol/L*: β = -0.11 (-0.16,-0.05), *p*<0.001 SNRPN methylation: *cord RBC folate 100 nmol/L*: β = -0.002 (-0.09,0.09), *p* = 0.96 IGF2 methylation: *cord RBC folate 100 nmol/L*: β = 0.11 (0.02,0.20), *p* = 0.02	
Voisin (2015), Greece [99]	Greek Healthy Growth Study, Obese: 35 (68) Normal weight: 34 (66)	24-hour recall for %energy from fat, cholesterol intake, MUFA/SFA, PUFA/SFA & MUFA+PUFA (~10y)	Infinium Human Methylation27 BeadChip	Blood	~10y	The methylation levels of one CpG island shore and four sites were significantly correlated with total fat intake. No significance was found for cholesterol intake. The methylation levels of 2 islands, 11 island shores and 16 sites were significantly correlated with PUFA/SFA; of 9 islands, 26 island shores and 158 sites with MUFA/SFA; and of 10 islands, 40 island shores and 130 sites with (MUFA+PUFA)/SFA Top 10 most significant CpG sites/islands (Gene, Coefficient, adjusted *p*: *%Energy from fat* *GPS1*: -0.0135, *p* = 0.006 *TAMM41*: 0.00987, *p* = 0.006 *TAS2R13*: -0.0118, *p* = 0.012 *MZB1*: 0.0145, *p* = 0.023 *TXNIP*: 0.0148, *p* = 0.043 *MUFA/SFA*: *ALDH3A2*: -0.289, p = 0.00097 *MYLK3*: -0.238, p = 0.00363 *LOC642852*: *–*0.317, p = 0.00364 *TPPP2*: *–*0.309, p = 0.00364 *RXFP2*: -0.262, p = 0.00364 *TMEM80*: -0.245, p = 0.00364 *SEMA3G*: 0.28, p = 0.00388 *VCAM1*: -0.259, p = 0.00482 *KRT73*: -0.245, p = 0.00496 *KRTCAP2*: -0.30, p = 0.0051 *PUFA/SFA* *CBR1*: 1.28, p = 4.02e–06 *RBCK1*: 0.687, p = *2*.*3e–05* *ABHD16A*: *-*0.302, p = 7.18e–05 *KRT23*: *-*0.326, p = 0.00536 *PDE3A*: *-*0.274, p = 0.0066 *NCOA1*: *-*0.42, p = 0.00722 *PCED1A*: *-*0.41, p = 0.00914 *MRPL13*: 0.308, p = 0.00914 *AKR7A2*: 0.237, p = 0. 00914 *FAM154A*:*-* 0.357, p = 0.0193 *(MUFA+PUFA)/SFA* *MRPL13*: 0.186, p = 0.000952 *NCOA1*: *-*0.233, p = 0.00308 *PCED1A*:- 0.213, p = 0.00308 *CCNA2*:- 0.126, p = 0.00308 *LCE1B*:- 0.254, p = 0.00352 *ALDH3A2*: -0.176, p = 0.00352 *MYLK3*: *-*0.166, p = 0.00352 *GBP7*: *-*0.175, p = 0.00352 *DGKI*:- 0.178, p = 0.00352 *DNTTIP*: 0.148, p = 0.00352	Tanner stage, cell-type composition
De La Rocha (2016), Mexico [100]	49 (55)	Serum fatty acids	Global DNA methylation using total 5-methyldeoxycytosine	Blood	Lactating infant (89.6±68.2d)	Change in %methylation per one % increase in FA *serum C20*:*4 (arachidonic acid)*: β = 0.08, *p* = 0.04 *serum C20*:*5 (eicosapentaenoic acid)*: β = 0.099, *p* = 0.04 No significant associations with other fatty acids (data not shown in main paper)	Age, birth weight, normalised weight gain
Lee (2012), US [26]	THREE, 141 (~47)	Serum copper,(87% GAD ≥37w)	*NFIX*, *FAPGE*, *MSRB3* using pyrosequencing	Cord blood	Birth	Association(95% CI) with serum copper ug/dl in cord blood: *NFIX*: β = 0.13 (0.06,0.20) *RAPGE*: β = -0.10 (-0.16,-0.05) *MSRB3*: β = -0.15 (-0.21,-0.08)	Batch effects
**Famine / Seasonality**							
Tobi (2015), The Netherlands [101]	Dutch Hunger Winter 885 (54) (Exposure during gestation:348,Periconceptional 74,Time-controls:160, Family-controls: 303)	Famine	Infinium HumanMethylation450 BeadChip	Blood	58.9±.5y	Famine vs. time-and family controls: % methylation (95%CI) Famine in 1–10 weeks gestation (n = 73) cg20823026 (*FAM150B/TMEM18*): 2.3 (1.5–3.1), p = 3.1x10^-8^ cg10354880 (*SLC38A2*):0.7(0.5,0.9), p = 5.9x10^-7^ cg27370573 (*PPAP2C*): 2.7(1.7,3.7), p = 3.6x10^-7^ cg11496778(*OSBPL5/MRGPRG*): -2.3(-3.1, -1.5), p = 2.1x10^-7^ Famine in 11–20 weeks gestation (n = 123): no significant cpgs Famine in 21–30 weeks gestation (n = 143): no significant cpgs Famine in 31- delivery (n = 128): no significant cpgs Any exposure to famine: cg15659713 (*TACC1*): 1.2(0.8,1.7), p = 2.0x10^-7^ cg26199857(*ZNF385A*): 2.0(1.3,2.7), p = 1.5x10^-7^ Conceived during extreme famine, but exposed for short period in gestation: cg23989336 (*TMEM105*):-3.5(-4.6,-2.3), p = 1.0x10^-7^	Age, sex, batch effects, cell heterogeneity, smoking status, current macronutrient and micronutrient intake and SEP
Finer (2016), Bangladesh [102]	143(58)	Famine (postnatal exposure 1-2y or exposure during gestation or unexposed)	Infinium HumanMethylation450 BeadChip 16 MEs: *VTRNA2-1*, *PAX8*, *PRDM9*, *HLA-DQB2*, *PLD6*, *ZFP57*, *AKAP12*, *ATP5B*, *LRRC14B*, *SPG20*, *BOLA*, *RBM46*, *ZFYVE28*, *EXD3*, *PARD6G*, *ZNF678*, *ZFYVE28*	Blood	Postnatal exposed: 31±0.4y Exposure during gestation:30±0.3y Unexposed: 28±0.3y	Postnatal exposure n = 49 vs gestational exposure n = 40 vs unexposed n = 54 Genome-wide analyses No differences between groups at 5% FDR Targeted DNA methylation Methylation differences between groups seen in 6/16 MEs at p<0.05, driven by gestational exposure group:: *VTRNA2-1*, *PAX8*, *PRDM9*, *ZFP57*, *BOLA*, *EXD3* z-score for mean methylation across all 16 MEs: gestational exposure: -0.24 postnatal exposure: -0.14 unexposed: -0.15 ANOVA *p* = 0.0003	Cell composition
Lumey (2012), The Netherlands [103]	Dutch Hunger Winter 947 (54) (Prenatal:350, Unexposed time controls:290, Unexposed same-sex sibling:307)	Famine	LINE-1 & Sat-2 using pyrosequencing Global methylation using LUMA	Blood	Prenatal exposure group:58.9 ±0.5y Unexposed time controls: 58.5 ±1.6y Unexposed same-sex siblings: 57.3± 6.3y	Changes in DNA methylation (%units) in exposed vs. all non-exposed: Global methylation: Mean % (SD): 75.2% (4.7) B(95% CI): -0.15 (-0.49, 0.81), p = 0.63 LINE-1 methylation % (SD): Mean % (SD):77.1% (2.5) B(95% CI): -0.05(-0.33, 0.22), p = 0.70 Sat2 methylation % (SD): Mean % (SD):122.2 (56.2) B(95% CI): -0.51 (-7.38, 6.36), p = 0.88	Age, within family clustering
Heijmans (2008), The Netherlands [104]	Dutch Hunger Winter 244 (~54) (periconceptional:60, late gestation: 62, Unexposed same-sex sibling:122)	Famine	*IGR2* DMR (5 CpGs) using mass spectrometry-based method	Blood	Periconceptional group: 58.1±0.35y Late gestation group: 58.8± 0.4y Controls: 57.1± 5.5y	Mean (SD) methylation in those periconceptionally exposed to famine vs. non-exposed siblings: *Average*: 0.488(0.047) vs. 0.515(0.055), p = 5.9x10^-5^ *CpG1*: 0.436(0.037) vs, 0.470(0.041), p = 1.5x10^-4^ *CpG2 and 3*: 0.451(0.033) vs. 0.473(0.055), p = 8.1x10^-3^ *CpG4*: 0.577(0.114 vs. 0.591(0.112), p = 0.41 *CpG5*: 0.491(0.061) vs. 0.529(0.068), p = 1.4x10^-3^ No difference in methylation of *IGF2* DMR between a subset exposed in late gestation and unexposed siblings	Age and family relations
Tobi (2014), The Netherlands [105]	Dutch Hunger Winter 48 (50)	Famine (early gestation)	1.2M CpGs using RRBS	Blood	58.1±0.35y	Genomic annotation-centred analysis of differential methylation after famine (vs. unexposed sibling), p_FDR:_ Genomic annotations** Non-CGI, ‘bona fide’ promoters: 0.026 Enhancers: 0.026 DNaseI/FAIRE-seq regions: 0.036 Middle exons: 0.036 Developmental enhancers type I: 0.036 ‘bona fide’ CGI shores: 0.053 Non-coding RNA: 0.053 Conserved regions: 0.053 CGI shores: 0.053 3’UTR: 0.085 Non genic CGI: 0.085 ‘Bonafide’ CGI border: 0.085 Developmental enhancer type II: 0.15 CGI: 0.15 Introns: 0.15 hESC bivalent chromatin domains: 0.28 Bonafide CGI: 0.32 Cell-type specific gene promoters: 0.32 First exons: 0.36 Promoters: 0.36 HSC bivalent chromatin domains: 0.36 Imprinted promoters: 0.36 ‘Bona fide’ CGI promoter: 0.37 CTCF insulators from CD4+ cells: 0.37 Imprinted DMRs: 0.37 Putative metastable epialles: 0.47 Variably methylated regions: 0.57 Promoters cancer genes: 0.63 Within the 5 annotations found to be significant, 181 DMRs were associated with prenatal famine p_FDR<0.05_	
Tobi (2009), the Netherlands [106]	Dutch Hunger Winter 244 (~54) (periconceptional:60, late gestation:62, unexposed same-sex sibling:122)	Famine	*GNASAS*, *GNAS* A/B, *MEG3*, *KCNQ1OT1*, *INSIGF* and *GRB10*, *IGF2R*, *IL10*, TNF, *ABCA1*, *APOC1*, *FTO*, *LEP*, *NR3C1* and *CRH* using mass-spectrometry based method	Blood	Periconceptional group: 58.1±0.35y Late gestation group: 58.8± 0.4y Controls: 57.1± 5.5y	Within-pair differences divided vs sibling controls, *p*: Periconceptional exposure *GNASAS*: 0.24, 3.1x10^-6^ *MEG3*: 0.21, 8.0x10^-3^ (non-significant after Bonferroni correction) *IL10*: 0.37, 1.8x10^-6^ *ABCA1*: 0.21, 8.2x10^-4^ *LEP*: 0.24, 2.9x10^-3^ *INSIGF*: -0.61, 2.3x10^-5^ Non-significant for all other loci Late gestation exposure: *No associations except for reduction in GNASAS*: -0.26, 1.1x10^-7^ Non-significant for all other loci	Family relatedness, bisulphite batch, age
Veenendaal (2012), The Netherlands [107]	Dutch Hunger Winter 759 (54%) (periconceptional:60, late gestation:62, unexposed same-sex sibling:122)	Famine	*PPARγ*, *GR1-C*, *PI3Kinase*, *LPL* using PCR	Blood	58±1y	Methylation differences % (95%CI) for exposed vs. unexposed: Late gestation: *GR*: 0.60 (-16.39, 21.05) *LPL*: 11.01 (-5.35, 30.34) *PI3Kinase*: 6.18 (-42.25, 95.03) *PPARγ*: -2.37 (-14.53, 11.52) Mid-gestation *GR*: -5.26 (-22.04, 15.14) *LPL*: 12.08 (-5.45, 32.84) *PI3Kinase*: *-32*.*36 (-64*.*33*, *28*.*27)* *PPARγ*: *-8*.*70 (-20*.*63*, *5*.*02)* Early gestation: *GR*: 6.82 (-15.55, 35.12) *LPL*: 9.20 (-10.95, 34.04) *PI3Kinase*: -40.84 (-72.56, 27.38) *PPARγ*:-6.76 (-21.08, 10.30) No significant associations were found	Maternal age, sex and parity
Waterland (2010), The Gambia [108]	The Keneba cohort 50 (50%) Conceived in rainy season:25, conceived in dry season:25	Famine	MEs: *BOLA3*, *FLJ20433*, *PAX8*, *SLOTRK1*, *ZFYVE28* using pyrosequencing	Blood	Conceived in rainy season:6.61±2.73y Conceived in dry season:7.05±2.67y	At all 5 MEs, DNA methylation was significantly higher among individuals conceived ruing the rainy season (i.e. hungry season): *BOLA3*: p = 0.03 *FLJ20433*: p = 0.03 *PAX8*: p = 0.02 *SLOTRK1*: p = 0.006 *ZFYVE28*: p = 0.002 *Overall*: p = 0.0001 Effect sizes were NR but highlighted as being large e.g. rainy season was associated with absolute methylation increments of over 10% at *PAX8* and *ZFYVE28*	

*Studies spanning more than one exposure may appear twice in the table;

** Abstract

AA: Age acceleration; ARIES: Accessible Resource for Integrated Epigenomic Study; BMI: Body Mass Index; BSCC: Bogotá School Children Cohort; CHO: Carbohydrate; CI: Confidence Interval; CBMCs: Cord Blood Mononuclear Cells; COBRA: Combined Bisulfite Restriction Analysis; D: Days; DMR: Differentially Methylated Region; DA: Dizygotic; FDR: False discovery rate; FFQ: Food frequency Questionnaire; GAD: Gestational Age at Delivery; HUVEC: Human Umbilical Vein Endothelial Cells; LUMA: Luminometric *methylation* assay); M: Months; MANOE: Maternal Nutrition and Offspring’s Epigenome Study; MoBA: Norwegian Moher and Child Cohort Study; MUFA: Monounsaturated fatty acid; MZ: Monozygotic; NEST: Newborn Epigenetics Study; NR: Not Reported; OR: Odds Ratio; PAH: Princess Anne Hospital Study; PETS: Peri/postnatal Epigenetic Twins Study PUFA: Polyunsaturated fatty acid; RBC: Red Blood Cell; SD: Standard Deviation; SE: Standard Error; SEP: Socioeconomic Position; SFA: Saturated Fatty Acid; THREE: Tracking Health Related to Environmental Exposures Study; W; Weeks Y: Year

**Table 3:** Socioeconomic position in early life and epigenetics. *(Organised by exposure*, *DNA methylation (epigenome wide*, *global methylation*, *imprinted genes*, *other genes)*.

	Cohort, N (% female)	Early life variable	DNA methylation	Tissue	Mean age at epigenetic measure ± SD (age range)	Main result	Confounders
Simpkin (2015), UK [58]	AIRES 1018 (51)	Maternal education	Infinium Human Methylation450 BeadChip to estimate Horvath epigenetic age	Cord blood & blood	Birth, 7y, 17y	ANOVA F-statistic & *p* for early life variable and age acceleration: Maternal education & AA at birth:0.55, *p* = 0.70 Maternal education & AA at 7 years: 0.37, *p* = 0.83 Maternal education & AA at 17 years: 1.40, *p* = 0.23 Longitudinal analysis of maternal education and & average AA during childhood CSE: ref Voc. -0.30(-1.84, 1.23) O-level: -0.24(-1.56, 1.08) A-level: -0.38(-1.69, 0.93) Degree: -0.51 (-1.87, 0.85) *p* = 0.76 Longitudinal analysis of maternal education and & changes in AA during childhood CSE: ref Voc. 0.11(-0.02, 0.24) O-level: 0.38(-0.07,0.15) A-level: 0.58(-0.05, 0.17) Degree: 0.10 (-0.01,0.22) *p* = 0.18	Cell-type proportions
Herbstman (2013), US [27]	CCCEH 279 (53.4)	Maternal education & maternal hardship (last trimester of pregnancy)	Global methylation using Methylamp Global DNA Methylation Quantification Kit	Cord blood & blood	Birth & ~3y	Maternal education & cord blood global methylation: *high school vs*. *no high school*: β = 0.10 (-0.29,0.50) *higher education vs*. *no high school*: β = 0.09 (-0.33,0.51) Material hardship (yes vs no) & cord blood global methylation: β = 0.09 (-0.23,0.42) Maternal education & 3y blood global methylation: *high school vs*. *no high school*: β = 0.03 (-0.41,0.48) *higher education vs*. *no high school*: β = -0.28 (-0.80,0.23) Material hardship (yes vs no) & 3y global methylation: β = -0.19 (-0.58,0.42)	GA, plate, maternal height, pre-pregnancy BMI, maternal age at delivery, ethnicity, sex, public assistance, total polycyclic aromatic hydrocarbons and environmental tobacco smoke
Perng (2012), Columbia [50]	BSCC 568 (53.7)	Maternal education, household socioeconomic stratum	LINE-1 using pyrosequencing	Blood	(5-12y)	LINE-1 methylation mean(SD) Maternal education (university): All:80.39 (0.70), p_trend_ = 0.34 Males: 80.71(0.56), p_trend_ = 0.06 Females: 80.13(0.72), p_trend_ = 0.78 Household socioeconomic stratum: All: 1(lowest): 80.35 (0.48) 2: 80.20 (0.67) 3: 80.21 (0.64) 4(highest): 80.62 (0.71) p_trend_ = 0.15 Males: 1(lowest): 80.40 (0.55) 2: 80.35 (0.68) 3: 80.32 (0.64) 4(highest): 80.62 (0.61) p_trend_ = 0.30 Females: 1(lowest): 80.29 (0.38) 2: 80.06 (0.63) 3: 80.13 (0.64) 4(highest): 80.62 (0.89) p_trend_ = 0.27	
Tehranifar (2013), US [113]	New York Women’s birth cohort, 90 (100)	Mother’s education, family income at birth	Sat2, Alu, LINE-1 using MethyLight	Blood	38-46y	Univariate analysis, methylation mean (95% CI) Sat2 *n* = 87 <high school: 92.5(83.6,101.3) ≥high school: 76.1 (67.6,84.7) *p*<0.05 Income Q1 (lowest): 97.0(83.6,110.3) 2:78.8 (66.4,91.1) 3: 83.2 (70.8,95.6) 4:72.9 (60.2,85.6) P<0.10 Alu *n* = 88 <high school: 108.8(97.4, 120.2) ≥high school: 105.8(94.4,117.3) p>0.05Income Q1 (lowest): 107.9(89.9, 125.8) 2: 110.9 (93.4,128.4) 3:103.4 (86.6, 120.5) 4: 112.3 (95.2, 129.4) P>0.05 LINE-1 *n* = 89: <high school: 167.2 (150.7,183.8) ≥high school:162.8 (146.5,179.2) p>0.05 Income Q1 (lowest):170.7(145.6,195.9) 2: 163.4(139.5,187.3) 3:155.3(131.4,179.2) 4:177.2(153.3,201.1) P>0.05 Multivariate association in Sat2 beta(95%CI), *n* = 73 Maternal education:3.4(-11.6,18.4) Lowest vs. highest Q: 22.5(0.8,44.1) Second vs. highest Q: 3.0(-16.9,22.9) Third vs. highest Q: 6.7(-11.5,24.8)	Age, prenatal smoke, birth order, adult education, adult occupation
King (2015), US [114]	NEST, 619 (NR)	Maternal education & income	DMRs in *IGF2*, *H19*, *MEG3*, *NNAT* using pyrosequencing	Cord blood	Birth	Maternal education, unstandardized β *p*, ref = 16y *IGF2*: 1-12y: -1.58, *p*<0.05 *p*<0.1 13-15y: -2.10, *p*<0.05 17+y: -1.74, *p*<0.05 *H19*: 1-12y: -1.16, NS 13-15y: -0.47, NS 17+y: -0.57, NS *MEG*: 1-12y: 0.53, NS 13-15y: -0.05, NS 17+y: -0.70, NS *NNAT*: 1-12y: -1.27, NS 13-15y: -1.13, NS 17+y: -0.28, NS Household income, unstandardized β *p*, ref = $100K *IGF2*: $25k:-1.19, NS $25-$50k: -1.87, *p*<0.1 $50-$100k: -0.89, NS *H19*: $25k:-1.07, NS $25-$50k: -1.10, NS $50-$100k: -0.94, NS *MEG3*: $25k:0.94, NS $25-$50k: -0.85, NS $50-$100k: 0.49, NS *NNAT*: $25k:1.37, NS $25-$50k: 0.96, NS $50-$100k: 1.78, NS	Mother and father's race, household income/education
Obermann-Borst (2012), The Netherlands [115],	HAVEN 120 (42)	Maternal education	*IGF2 DMR*, *IGF2R*, *INSIGF* using PCR	Blood	17±2.5m	β(SE) for change in methylation from linear mixed model *IGF2* DMR: -0.3(0.9),p = 0.71 *IGF2R*: 2.4(1.5), p = 0.11 *INSIGF*: 1.4(0.6), p = 0.02	Correlation between individual CpG dinucleotides, bisulfite batch, smoking
Soubry (2011), US [38]	NEST, 436 (47.5)	Maternal education	*IGF2* DMR (3 CpGs) and *H19* DMR (4 CpGs) using pyrosequencing	Cord blood	Birth	Mean methylation %(SD), difference (*p*) *IGF2*: *College yes*: 46.99 (6.61) *College no*: 47.72 (7.04) Δ: -0.73 (0.34) *H19*: *College yes*: 58.90 (7.45) *College no*: 60.73 (8.07) Δ: 1.83 (0.03)	
Obermann-Borst (2013), The Netherlands [64]	120 (42)	Maternal education	*LEP* using mass-spectrometry based method	Blood	17±2.5m	% Absolute methylation change (SE) & % Relative methylation change (SE) from linear mixed model *Model 1* low education: 2.1 (0.8)*;+*9.1(3.5), *p =* 0.008 *Model 2* low education: +1.0 (0.8); +4.2 (3.4), *p* = 0.23	Model 1 Correlation between individual CpG dinucleotides, bisulfite batch, GA Model 2 Correlation between individual CpG dinucleotides, bisulfite batch, smoking, breastfeeding, sex, birthweight, BMI serum leptin
Wijnands (2015), UK [98]	120 (41.7)	Mother’s education	*LEP* & *TNFα* using mass-spectrometry based method	Blood	17±2.5m	*TNFα* & *LEP* methylation were not associated with maternal education	
Mulligan (2012), Democratic Republic of Congo [46]	25 (NR)	Maternal deprivation	*NR3CI* (39 CpGs) using PCR	Cord blood	Birth	First PC of % methylation of 39 CpG sites explained 16.15% of variance & correlated with material deprivation r = 0.44, p = 0.03	
Agha (2014), US** [116]	New England Family Study birth cohort, 106 (64)	Parental SEI	Infinium Human Methylation450 BeadChip	Subcutaneous adipose tissue & peripheral blood leukocytes	44-50y	Adipose tissue Parental SEI was associated with DNA methylation in women (p <0.001), but not men or the pooled sample. Blood Parental SEI was not related to blood DNA methylation	Race, smoking, mother’s smoking during pregnancy
Terry (2008), US[60]	92 (100)	Family SES (measured by parental education and income at birth and 7y)	Global DNA methylation using [3H]-methyl acceptance assay	Blood	42.28y	Multivariate linear regression DPM/μg(95% CI) for association between DNA methylation by variables *Family SES*: -0.01 (-0.01,0.002)	Smoke exposure, adult BMI, race, birth weight, age at menarche, childhood passive smoking, parity, age at first birth
Beach (2016), US [117]	398 (55)	Preadolescent cumulative SEP risk (11.7y)	Infinium Human Methylation450 BeadChip	Blood	19.3y	28,640 loci were associated at the *p*<0.01 level of significance, with 2,032 loci associated at FDR<0.05. No specific loci presented	Sex, age
Lam (2012), Canada [118]	92 (62)	Early life SES	Infinium Human Methylation27BeadChip	Blood	33.04±5.03y	3 differentially methylated CpGs (<5% change were found comparing low SES n = 46) with high SES (n = 46). Individual effect estimates NR	
Borghol (2011), UK [119]	1958 British Birth cohort 40(0)	Cumulative SEP Index	Genome-wide methylation (MeDIP)	Blood	45y	3112 probes (6176 genes) were variably methylated when comparing SEP extremes. Unsupervised hierarchical cluster was applied to the 500 most variables probes. A large cluster was found to be enriched with high SEP individuals. 1252 gene promoters associated with childhood SEP were identified	
Subramanyam (2013), US [120]	MESA 988 (52)	Childhood SES	LINE-1 and Alu using pyrosequencing	Blood	44-84y	Mean difference (SE) in DNA methylation per category change in exposure (low, medium, high) *LINE-1*: 0.04(0.06), p>0.05 *Alu*: 0.02(0.05), p>0.05	Age, sex, race
Beach (2014), US [121]	388 (55)	Preadolescent cumulative SEP risk 11.7y	*SLC6A4* (16 CpGs) measured using Infinium Human Methylation450 BeadChip	Blood	19.3y	*P* from two-way ANOVA. ** indicated significant after multiple testing cg12074493 *p* = 0.588 cg06841846 *p* = 0.198 cg18584905 *p* = 0.494 cg27569822 *p* = 0.816 cg10901968 *p* = 0.241 cg26741280 *p* = 0.138 cg25725890 *p* = 0.500 cg05016953 *p* = 0.922 cg14692377 *p* = 0.001** cg03363743 *p* = 0.322 cg22584138 *p* = 0.502 cg05951817 *p* = 0.555 cg26126367*p* = 0.139 cg01330016 *p* = 0.032 cg24984698 *p* = 0.006** cg20592995 *p* = 0.640	

**Abstract;

AA: Age acceleration;ARIES: Accessible Resource for Integrated Epigenomic Study; BSCC: Bogotá School Children Cohort;CCCEH: The Northern Manhattan Mothers and Newborns Study of the Columbia Center for Children’s Environmental Health;DMR: Differentially Methylated Region; DPM: Disintegrations Per Minute; M: Months; MeDIP: Methylated DNA Immunoprecipitation; MESA: The Multi-Ethnic Study of Atherosclerosis; NEST: Newborn Epigenetics Study; NR: Not reported; PC: Principle Component; SEI: Socioeconomic Index; SEP: Socioeconomic Position; SES: Socioeconomic Status; Y: Years

### Body size and growth in early life

Of the included papers, *n* = 56 examined the role of body size and growth in early life on DNAm (Table 1). There were 14 prospective (3 of which compared extreme groups), 33 cross-sectional (6 of which compared extreme groups), and 9 twin studies.

#### Prospective studies of body size and growth in early life and DNA methylation

Thirteen prospective papers examined size at birth [53, 55, 56, 58–62, 64–66, 77, 78], one paper body size in childhood [58], and two growth [59, 67].

Body size at birth: Three papers examined body size at birth in relation to childhood and adolescent genome-wide methylation using the Illumina Human-Methylation450 or Human-Methylation27 BeadChip array [55, 58, 77]. Agha *et al*. demonstrated that birth weight-for-gestational age (GA) was associated with methylation at 34 CpGs of which 4 of these CpGs remained at age 7–10 years in 235 children. Three of these CpGs were located on *PBX1* (embryonic development regulator) and one was on *NOS1AP* (neuronal nitric oxidase synthase) [55]. In the Accessible Resource for Integrated Epigenomic Studies cohort (ARIES, a sub sample of The Avon Longitudinal Study of Parents and Children (ALSPAC) cohort), birth weight was not associated with genome-wide DNA methylation in blood when the children were aged 7 and 17 years old [77]. However, analyses in the ARIES cohort did find that birth weight was associated with age acceleration based on Horvath’s clock (i.e. residuals from regression of epigenetic age on actual age) at birth, 7 and 17 years; a finding that was replicated in an independent cohort [58].

Two studies examined associations between body size at birth and global DNA methylation in adulthood [59, 60]. Rerkasem *et al*. found no associations between birth weight or birth length and blood methylation at LINE-1 or Alu in 249 20 year old adults [59]. In the other paper, global methylation measured in blood at age 38–48 years using a [^3^H]-methyl acceptance assay, was associated with birth length, but not birth weight [60].

Five papers examined body size at birth and subsequent DNAm in candidate genes [53, 61, 62, 64, 65]. Three of these papers examined methylation in imprinted genes. In the Motherwell Cohort, there was an association between birth length, but not birth weight, and methylation at *IGF2/H19* differentially methylated region (DMR) measured in blood at 40 years [61]. Birth weight was associated with *H19* DMR measured in childhood (~8 years) in girls, but not boys [53] and with methylation at the *IGF2* DMR measured in blood samples of infants aged 17 months [62]. In relation to non-imprinted genes, birth weight was found to be associated with methylation at *HSD2*, but not *GR* (both related to glucocorticoid) in blood samples of 34 participants aged 40 years [61] while another study found it not to be associated with methylation at the *LEP* gene in blood among infants aged ~17 months once confounders were taken into account [64].

Among the papers comparing extreme groups, one found no genome-wide differences in DNA methylation in adipose derived stem cells between 13 low birth weight (LBW) babies and controls [56]. Another found some evidence for a difference in methylation in specific CpG sties of *IGF2* in blood between 158 very LBW (≤1500g) with controls [63]. The third paper found that methylation at two out of three CpG sites in *ACE* (angiotensin-converting enzyme, a gene related to cardiovascular disease) was lower among LBW children (6-12y) compared with normal birth weight children [79].

Childhood body size and growth: Using data from the ARIES cohort weak, associations between taller height at 7 years and epigenetic age acceleration at 7 and 17 years (*p =* 0.06, *p* = 0.07) were observed. However, no associations were seen with BMI [58].

Two papers examined growth in early life in relation to DNA methylation [59, 67]. In one, catch up growth during the first year of life was associated with Alu but not LINE-1 methylation measured in blood at 20 years [59]. In the other, those defined as rapid growers between term and 12 weeks had higher methylation at *TACSTD2* (associated with adiposity) at 12 years compared with slow growers. This was not replicated in ALSPAC where methylation was measured at 7 years [67].

#### Cross-sectional studies of body size and growth in early life and DNA methylation

Most (n = 28) of the cross-sectional papers investigated the association between birth weight and cord blood DNA methylation (Table 1). Five included birth length/head circumference/crown heel length [27, 43, 80–82], one body composition at birth [82], and six childhood height/weight [49, 50, 52–54, 83].

Birth weight: Five papers examined birth weight and cord blood genome-wide methylation measured using the Illumina Human-Methylation450 or Human-Methylation27 BeadChip array [21, 22, 24, 25, 55]. In a Norwegian study, birth weight was associated with methylation at 19 CpG sites including CpGs on the *ARID5B* and *XRCC3* genes which are related to adipogenesis and DNA repair respectively [21]. Birth weight percentile also related to methylation in three genes of which one, *FGFR2* (involved in metabolic regulation) replicated in a cohort of 110 participants [22]. Fryer *et al*. observed 304 CpG sites to be associated with birth weight percentile in 12 newborns [25]. However no genome-wide significance between birth weight and cord blood methylation was found among 201 participants of another study [24]. Using a different microarray technique, Lee *et al*. found birth weight to be associated with differentially methylated regions (DMRs) near three genes involved in early development (*NFIX*, *RAPGEF2*, *MSRB3*) [26].

Six papers examined markers of global methylation in cord blood [27–30, 32, 84]. There was no evidence for an association between birth weight and cord blood global methylation measured using Methylamp, LUMA, LINE-1 or Alu in most papers [27, 28, 32, 84]. One paper observed an association (*p* = 0.05) between lower birth weight and higher cord blood LINE-1 methylation,[29] while others found that LINE-1 methylation was slightly lower among newborns with high birth weight compared with normal weight [30].

The remaining papers examined cord blood methylation in candidate genes with the majority focused on imprinted genes. Five reported associations with cord blood methylation at imprinted genes in the Newborn Epigenetics Study (NEST) [35–38, 40]. Most did not demonstrate an association between birth weight and *IGF2* methylation [35, 36, 38]. However, one observed a lower methylation at *IGF2* DMRs among low birth weight compared with normal weight newborns (p = 0.06) [37]. There was a significant relationship between birth weight and methylation at *PEG10* and/or *PLAGL1* in three NEST papers [35, 37, 40]. Findings for *H19* methylation were inconsistent [35, 36, 38]. In another study, methylation at *IGF2* was lower in high birth weight newborns compared with normal birth weight groups [39]. There was no correlation between birth weight and methylation at the *ZAC1* DMR [43] or with methylation of *IGF2*, *H19*, *PEG3*, *SNRPN* [29, 32].

In the papers investigating non-imprinted genes, birth weight was not associated with methylation in genes related to the glucocorticoid receptor [32, 46]. A follow on study from the paper by Fryer *et al*. [25], found that increased cord blood methylation at *GSTM5* and *MAP2K3* was associated with a reduced risk of a lower birth weight percentile while higher methylation levels in *APOB* were associated with an increased risk [45]. Birth weight was also associated with *AHRR* (involved in cell growth and differentiation), *HFI3A (*obesity-associated gene*)* and *LEP* (appetite-related) methylation [44, 47, 48].

Among the papers comparing extreme groups, Qian *et al*. did see differences in the methylation of *H19*, but not *MEST*, in cord blood between 39 small–for-GA (SGA) versus average-for-GA (AGA) babies [34]. Similarly, Zhang *et al*. found methylation at *H19* DMR in blood to be different between AGA, SGA and large-for-GA infants [41].

Other body size measures at birth: There was no evidence for an association between birth length, ponderal index, head circumference, crown heel length and global cord blood methylation [27–29, 43, 47], at imprinted genes [29, 43] or *HIF3A* [47].

Among 991 participants of Chinese, Malay or Indian ethnicity, subscapular skinfold thickness and subscapular:triceps skinfold thickness increased with increasing methylation at 2 CpG sites in *HIF3A* [47].

Childhood height/weight: In school age children (5-12y) in Columbia (n = 568), there was no association between global blood DNA methylation and height-for-age z-score [50]. Methylation in 4 out of 8 CpG sites at the P2 promoter region of *IGF1* was inversely correlated with height in both a discovery and replication cohort [52]. There was no difference in the methylation of *H19* DMR comparing overweight versus lean boys or girls aged ~8 years [53]. Among 64 African-American children (5-6y), there was a weak association between lower BMI percentiles and higher saliva methylation in *DNMT3B*, but no relationships with other obesity-related genes (*FTO*, *MAOA*, *SH2B1*, *LEPR*, *BDNF* or *CCKAR*) [54]. Ouni *et al*. identified differently methylated CpG sties in *IGF* promoters between 94 children (~10y) with idiopathic short structure compared to children of normal height [51].

#### TWIN-studies of body size and growth in early life and DNA methylation

All twin studies examined birth-weight discordance [68–76]. There were no genome-wide DNAm differences between birth weight-discordant monozygotic (MZ) twins in blood from adults in two papers [68, 69], or using saliva samples from 15 year olds in another [71]. In twin participants aged 22–45 years, although 45 differentially methylated CpGs were identified using saliva samples, there was no difference in the methylation of repetitive elements [75]. In TwinsUK, one CpG of *IGF1* was associated with birth weight discordance [70] while there was a 13% average difference in methylation of *COMT* (implicated in psychiatric disorders) between MZ twins at 5 years [76].

### Nutrition in early life

Thirty seven papers included in this systematic review examined the role of nutrition in early life (Table 2). The majority of these studies (37%, *n* = 14) investigated maternal nutrition during pregnancy as a proxy for fetal nutrition. Nine studies examined nutrition in early life and six studies looked at both maternal pregnancy and early life nutrition. We also included eight studies that examined the impact of gestational exposure to famine or periods of restricted dietary intake

#### Maternal nutrition during pregnancy and offspring DNA methylation

Most papers focused on the nutrients involved in one-carbon metabolism i.e. folate, vitamin B6, vitamin B12, methionine, choline, and betaine given their role as methyl donors [14].

Nutrition-related methyl donor intake and/or supplementation: Joubert *et al*. identified 443 CpG sites, measured on the Illumina Human-Methylation450 BeadChip, that were differentially methylated in cord blood in relation to maternal plasma folate [85]. No association was observed in three of the four papers which examined maternal nutrition-related methyl donor intake/folic acid supplementation in relation with infant cord blood global DNA methylation [81, 86–88]. The forth paper found an inverse association between folic acid supplementation after 12 weeks gestation and LINE-1 methylation [81].

Six papers examined imprinted genes. Hoyo *et al*. found no differences in cord blood *IGF2* methylation among infants born to women taking moderate to high (≥400 μg/d) folic acid supplements before or during pregnancy compared to non-users, however *H19* methylation was reduced [90]. While Loke *et al*. also observed a reduction in infant’s *H19* methylation, they found an increase in one *IGF2* DMR (DMR2) across different tissues for mothers taking folic acid [91]. Similarly mean blood *IGF2* methylation of 17 month old infants was higher in those whose mothers took folic acid [62]. Another paper found that methylation at *ZAC1* was positively correlated with maternal intakes of vitamin B2 prior to pregnancy, however no association was observed for any other B-vitamin intake or folic acid supplement [64].

Two papers considered the effect at other candidate genes. In one, a difference in cord blood methylation at *LEP*, *RXRA* and/or *DNMT1* was observed for the intake of certain methyl donors [87]. However there was no association between maternal folic acid supplementation and blood *LEP* methylation among 17 month old infants in the other [64].

Nutrition-related methyl donor biomarker:In the paper by Haggarty *et al*., maternal red blood cell (RBC) folate was inversely associated with LINE-1 methylation [81]. Similarly, another paper observed that maternal serum markers of vitamin B12 were correlated with cord blood global DNA methylation [89]. Results from four papers examining maternal methyl donor biomarkers in relation to offspring’s cord blood methylation at imprinted genes were inconsistent [35, 93, 94]. In the Gambian Keneba cohort, serum vitamin B2, vitamin B6, homocysteine, and cysteine were associated with methylation at the combined metastable epialleles (MEs i.e. alleles that are variably expressed in genetically identical individuals due to epigenetic modifications [109])) [95].

Other nutrient intake/biomarker:Four papers investigated the effect of maternal intake of other nutrients. One found no association of maternal intake of protein, fat or carbohydrate with LINE-1 or Alu methylation [59]. Findings from the Motherwell cohort suggest that higher maternal intake of meat/fish and vegetable and lower intake of bread/potato in late pregnancy is associated with methylation at *HSD2* and *GR* in adult offspring blood [61], while another observed that lower maternal carbohydrate intake, but not fat or protein, was associated with higher cord blood methylation of *RXRA* but not of *eNOS* [96]. Finally, Simpkin *et al*. observed an association with maternal serum selenium, but not vitamin D, in children ages 7 and 17 years [58].

#### Early life nutrition and offspring DNA methylation

Breastfeeding:Five papers examined the impact of breastfeeding on DNA methylation. In Simpkin *et al’s* epigenetic age paper there was no correlation with breastfeeding duration [58]. In secondary analyses in another paper there was an implied association between breastfeeding and DNA methylation at approximately 11 years as measured on the Illumina Human-Methylation27 BeadChip, however no statistical test was performed [97]. In two papers using the same sample of 17 month old infants, there was a reduction in blood methylation of *LEP* with increasing duration of breastfeeding [64, 98]. A correlation between breastfeeding and methylation of a cancer-related gene, *CDKN2A*, in tumour tissues among premenopausal but not postmenopausal women was observed in the final paper [65].

Nutrition-related methyl donor biomarker:Seven papers examined the role of early life nutrition-related methyl donor biomarkers [25, 31, 50, 81, 88, 89, 93]. Across three cross-sectional papers, plasma homocysteine concentrations were negatively correlated with cord blood LINE-1 methylation or were different between two clusters defined by unsupervised hierarchical clustering using data from the Illumina Human-Methylation27 BeadChip [25, 31, 88]. In the Haggarty *et al*. paper described above, authors also observed that RBC folate in cord blood was associated with cord blood LINE-1, and methylation in *IGF2*, *PEG-3* but not *SNRPN* [81]. However, serum folate/plasma B12 was not cross-sectionally associated with cord blood LINE-1 methylation or blood samples of 5–12 year olds in two studies [50, 88]. While a negative cross-sectional correlation between serum B12 and *IGF2* cord blood methylation was observed in one study [89], this was not replicated by Ba *et al*, who also found no correlation with folate [93].

Other nutrient intake/biomarker:One paper found that fatty acid intake was associated with methylation levels in children’s blood as measured by from Illumina Human-Methylation27 BeadChip [99]. Another observed an association between HDL-cholesterol, but not LDL-cholesterol, and blood methylation at *LEP* and *TNF*α among young children. However this was attenuated after Bonferroni correction [98]

Two cross-sectional studies examined the effect of other early life nutrient biomarkers. One observed an association with arachidonic acid and eicosapentaenoic acid, but not other fatty acids in lactating infants global blood methylation [100]. The other paper reported an association between serum copper and *NFIX* but not *FAPGE* or *MSRB3* cord blood methylation [26].

#### Famine/rainy season exposure and offspring DNA methylation

The Dutch Hunger Winter, which lasted from September 1944 to May 1945, was the setting for 75% of the famine papers [104, 106, 107, 110–112]. In these papers DNA methylation was measured in blood samples of adults with mean age of 59 (0.5 SD) years who were exposed to famine at some point during gestation and compared with time and/or family matched controls. Using the Illumina Human-Methylation450 BeadChip, famine exposure during gestational weeks 1–10, but not later, was associated with differences in DNA methylation [105]. This time-sensitive association was also seen for *IGF2* methylation [104], and in an investigation of 15 candidate genes that are involved in metabolism, CVD and growth [106]. However, one study did not find an association between famine exposure at any point in gestation and DNA methylation at genes involved in stress response, developmental process and lipid metabolism [107].

Two papers were from other settings. In a sample of children in rural Gambia, methylation at MEs was higher among children conceived during the rainy season (i.e. “hungry” period) compared with those conceived in the dry season [108]. In Bangladeshi young adults no genome-wide differences in methylation was observed between those postnatally exposed to famine, exposed during gestation or unexposed [102]. However, a difference in methylation at MEs between those exposed to famine during gestation compared to the other groups was found [102].

### Socioeconomic position in early life

17 papers investigated the association between markers of SEP and DNA methylation (Table 3).

Maternal education: There was no association between maternal education and epigenetic age acceleration in the Simpkin *et al* paper [58] and no association with global methylation in two other papers [27, 50]. Tehranifar *et al*. found no association with LINE-1 or Alu methylation, but did observe higher blood Sat2 methylation among adults who’s mother’s had lower education compared with those whose mothers had at least a high school education [113]. Although one study found that maternal education was associated with cord blood *IGF2* methylation, but not with other imprinted genes [114], two other papers did not observe an association with *IGF2* methylation [38, 115]. However, in one of these papers an increase in *H19* methylation in cord blood of those with mothers who did not have a college education was reported [38].

In three papers using the same sample of 120 children aged 17 months, maternal education was correlated with *INSIGF* but not with *LEP* or *TNFα* blood methylation [64, 115, 122].

Other markers of SEP: No association was observed between family SEP measured by parental education and income at birth and 7 years, and blood measures of global DNA methylation in adults [60]. In a Columbian cohort of children aged 5–12 years, household socioeconomic stratum was not associated with blood LINE-1 methylation [50]. King *et al*. found that household income was associated with methylation at *MEG3* in cord blood, but not with other imprinted genes [114]. Results from a peer-reviewed abstract suggested that parental SEP was associated with DNA methylation in adipose tissue, but not blood of adult women as measured by Illumina Human-Methylation450 BeadChip [116].

Two papers using the same sample found preadolescent cumulative SEP risk (measured by family poverty, primary caregiver education, primary caregiver unemployment, single-parent family, receipt of assistance, and income) to be related to 2,032 loci at false discovery rate (FDR) <0.05 using data from the Illumina Human-Methylation450 BeadChip [117] and to specific CpG sties in *SLC6A4* [121].

Lam *et al*. used the Illumina Human-Methylation27 BeadChip to find three differentially methylated CpGs between adults with low early life SEP as defined by their parents occupation compared with high SEP [118]. Similarly, using a genome-wide approach, Borghol *et al*. found that childhood SEP as measured by father’s occupation and access to household amenities, was associated with methylation at 1,252 gene promoters in blood measures of 45 year old adults [123]. In the multi-ethnic study of atherosclerosis study, there was no evidence for an association between childhood SEP and LINE-1 and Alu blood methylation in adulthood [120].

## Discussion

This systematic review identified 90 papers that examined the relationship between body size, nutrition and/or SEP in early life with epigenetic markers measured at the same time or after the exposure. DNAm was the epigenetic marker used in all of the included studies. There was no strong evidence for a consistent association between these early life variables and DNAm. This may be due to the heterogeneous study designs, data collection methods and statistical analyses. Despite these inconclusive results, the hypothesis that the early life environment can impact DNAm, potentially persisting into adult life, was supported by some studies and warrants further investigation.

There has been one previous non-systematic review examining the impact of body size, and/or nutrition and SEP on DNAm [15] and one systematic review examining the effect of breastfeeding [16]. Our search strategy was designed to be sensitive; therefore we captured a large number of initial papers and included substantially more papers than the previous reviews. We limited results to articles published in English which may have excluded relevant non-English language papers There were slight differences in the papers included in our systematic review compared with previous reviews. For instance, Demetriou *et al*. included RCTs and studies where DNAm was the exposure. Hartwig *et al*. included animal studies and studies of methQTLs. However, our overall conclusions are in line with these reviews.

Of the three exposures (body size, nutrition and SEP) examined in this review, the majority of papers investigated body size in early life particularly birth weight. Birth weight can be considered as a proxy for the *in utero* environment, which may subsume maternal diet and parental SEP. This time in the life course marks a period of rapid development during which epigenetic processes, including DNAm are becoming established [10]. Therefore, it is no surprise that this sensitive time period has been the subject of the majority of epigenetic studies to date. However, the results from these studies have been inconsistent and the direction of the association, particularly in cross-sectional studies, remains unclear. One of the interesting findings from the Dutch Hunger Famine study is that nutritional insults in early gestation are more sensitive to lasting changes in DNAm compared with later gestation. Using birth weight as a proxy for the entire gestational period may mask these time-specific effects. There are fewer studies on the impact of post-natal body size, nutrition and SEP. There is some weak and inconsistent evidence to support the impact of body composition, childhood body size, breastfeeding, intake and biomarkers of nutrition related methyl-donors in early life as well as SEP on DNAm that can last into later life. There is also evidence from intervention studies suggesting folic acid and fish oil supplementation during pregnancy or early life results in changes in DNAm [124–126], which were outside the scope of our review.

The inability to come to a conclusive interpretation based on studies in this systematic review is due to extensive heterogeneity in the study designs, statistical analyses and small sample sizes. This is no surprise given that the field of epigenetics in relation to life course epidemiology is in its infancy. Since DNAm can be influenced by stochastic, genetic and environmental exposures, effect sizes, even if they represent causal effects, are likely to be small and therefore difficult to find in small studies [11]. The sources of heterogeneity common to other systematic reviews of observational studies are a concern here. For example, there is inconsistency in how exposures were recorded or measured between the studies which may have introduced heterogeneity. Similarly, not all studies adjusted for the same confounding factors, nor are we clear about what those confounders should be. Of particular concern is the oversight of some relevant studies to control for maternal smoking which is to date the strongest known environmental exposure to impact DNA methylation [127], and cellular heterogeneity [128]. Another source of heterogeneity is the method through which studies account (or do not account) for multiple testing with some studies using a Bonferroni correction and others using false discovery rate. It has been argued that using a Bonferroni correction in epigenome wide association studies may be too conservative due to potential patterns of co-methylation [129]. However, the potential for false positives makes for cautious interpretation of any positive findings in studies which don’t account appropriately for multiple testing. In addition, reproducibility of these findings will be an important goal for future research [128]. One of the unique characteristics of studying DNAm compared to genetics is that DNAm is tissue-specific [128]. The majority of studies included in this review have examined blood due to the ease of accessibility. It may be the case that the impact of e.g. nutrition in early life on DNAm may be more evident in adipose or other target tissues compared with blood.

A major limitation of all the studies is that knowledge of the epigenome, and DNAm, is still limited [128]. Most of the studies included in this review have focused on candidate genes, similar to how early genetic studies were carried out. A variety of assays were used to measure DNAm, which have been discussed in previous papers [129, 130]. As technology has advanced, the study of genome-wide methylation has increased. However, even the relatively advanced methods such as Illumina 450k (or the new 850k) covers an estimated <2% of the epigenome [128]. This implies that sites of interest may be missed. These technological issues have been discussed extensively by Mill and Heijmans [128].

In addition to these statistical and technological issues, interpreting the functional consequences of some of the identified DNAm sites remains relatively unexplored, as is the potential impact of these DNAm changes on phenotypic health outcomes. A recent paper from the Dutch Hunger Famine study providing evidence that DNAm may mediate the link between adversity in early life and health outcomes in adulthood is one of the first to support this hypothesis [131].

In light of findings from this review and suggestions from previous commentaries [128, 132, 133], we propose the following recommendations for future studies: 1) use of longitudinal studies to assess the impact of early life environmental exposures on the dynamics of the epigenome through the life course 2) full consideration of statistical issues, such as adjustment for confounding, ensuring sufficient power, control for multiple testing, and reproducibility 3) control for cell heterogeneity and examine associations across different tissue types 4) assess the functional consequence of identified epigenetic marks through second-generation EWAS as part of an integrated functional genomics strategy 5) examine if DNAm mediates the relationship between early life exposures and health outcomes in later life and use of novel methods to assess causality e.g. Mendelian Randomisation.

Overall, evidence for the impact of body size, nutrition and/or SEP in early life on concurrent or subsequent DNAm is inconclusive. However, findings to date are supportive of the continued investigation using well designed studies which capitalise on emerging technologies to test these hypotheses. Whether these early life-mediated DNAm profiles translate into health outcomes in later life is something that should be incorporated into future studies.

## Supporting information

S1 Table Search terms. (DOCX)

S2 Table PRISMA 2009 checklist. (DOC)
