# Supplementary material for: Associations between body size, nutrition and socioeconomic position in early life and the epigenome: A systematic review
Source: PLoS One. 2018 Aug 10;13(8):e0201672. doi: 10.1371/journal.pone.0201672 (PMC6086410; doi:10.1371/journal.pone.0201672)
Supplement: S1 Table — (DOCX) [file pone.0201672.s001.docx]

| **Supporting Information**  **S1 Table. Search terms** | |
| --- | --- |
| **Epigenetics** | |
| 1 | (epigen* ) |
| 2 | (EWAS) |
| 3 | (Methylation) |
| 4 | (DNA adj5 methyl*) |
| 5 | (differential adj1 methylation) |
| 6 | (global adj1 methylation) |
| 7 | (DNA adj5 hypermethyl*) |
| 8 | (DNA adj5 hypomethyl*) |
| 9 | (gene* adj5 methyl*) |
| 10 | Gene Expression Regulation |
| 11 | (methyl* adj5 region) |
| 12 | (histone adj1 modif*) |
| 13 | 1 OR 2 OR 3 OR 4 OR 5 OR 6 OR 7 OR 8 OR 9 OR 10 OR 11 OR 12 |
| **Body size/ growth** | |
| 14 | ((premature or pre-mature or preterm or pre-term) adj2 (birth or infant* or child* or neonat*)) |
| 15 | (gestation* adj2 age) |
| 16 | (weight or body-size or bmi or height or length or head-circumference) |
| 17 | ((grow*) adj3 (fet* or neonat* or prenatal* or pre-natal* or intrauterine or in-utero or postnatal or post-natal or birth* or infant* or child* or early-life or earlylife)) |
| 18 | (fet* or neonat* or prenatal* or pre-natal* or intrauterine or in-utero or postnatal or post-natal or birth* or infant* or child* or early-life) |
| 19 | 16 OR 17 |
| 20 | 18 AND 19 |
| 21 | 14 OR 15 OR 20 |
| **Nutrition** | |
| 22 | (nutrition adj3 (maternal or mother* or pregn* or fet* or neonat* or prenatal* or pre-natal* or intrauterine or in-utero)) |
| 23 | ((nutrition or diet or wean*) adj3 (postnatal or post-natal or infant* or child* or early-life)) |
| 24 | (breastfe* or breast-fe*) |
| 25 | (formula adj3 (infant* or child* or early-life)) |
| 26 | 22 OR 23 OR 24 OR 25 |
| **Socioeconomic position** | |
| 27 | ((occupation* or education*) adj3 (father* or mother* or parent*)) |
| 28 | ((income or manual) adj3 (father* or mother* or parent*)) |
| 29 | ((social class or social status) adj3 (father* or mother* or parent* or child* or early-life)) |
| 30 | ((socioeconomic or socio-economic) adj3 (father* or mother* or parent* or child* or early-life)) |
| 31 | ((deprivation or poverty) adj3 (child* or early-life)) |
| 32 | ((overcrowding) adj3 (child* or early-life)) |
| 33 | 27 OR 28 OR 29 OR 30 OR 31 OR 32 |
| **Combining results** | |
| 34 | 21 OR 26 OR 33 |
| 35 | 13 AND 33 |
